# Isomerization of Internal Alkynes to Iridium(III) Allene Complexes via C–H Bond Activation: Expanded Substrate Scope, and Progress towards a Catalytic Methodology

**DOI:** 10.3390/molecules201119686

**Published:** 2015-11-10

**Authors:** Neha Phadke, Michael Findlater

**Affiliations:** Department of Chemistry and Biochemistry, Texas Tech University, Lubbock, TX 79409-1061, USA; neha.phadke@ttu.edu

**Keywords:** iridium, allenes, pincer, isomerization, synthesis, isolation, crystal structure

## Abstract

The synthesis of a series of allene complexes (POCOP)Ir(η^2^-RC==CR’) **1b**–**4b** (POCOP = 2,6-bis(di-*tert*-butylphosphonito)benzene) via isomerization of internal alkynes is reported. We have demonstrated that the application of this methodology is viable for the isomerization of a wide variety of alkyne substrates. Deuterium labeling experiments support our proposed mechanism. The structures of the allene complexes **1b**–**4b** were determined using spectroscopic data analysis. Additionally, the solid-state molecular structure of complex **2b** was determined using single crystal X-ray diffraction studies and it confirmed the assignment of an iridium-bound allene isomerization product. The rates of isomerization were measured using NMR techniques over a range of temperatures to allow determination of thermodynamic parameters. Finally, we report a preliminary step towards developing a catalytic methodology; the allene may be liberated from the metal center by exposure of the complex to an atmosphere of carbon monoxide.

## 1. Introduction

The C-H bond is pervasive in organic molecules and activation of this bond has been intensely studied [1]. A variety of methods have been developed for this purpose: free-radical halogenation, electrophilic aromatic substitution, and deprotonation of acidic C–H bonds are early examples of C–H functionalization. The potential to drastically simplify and shorten synthetic sequences has driven the continued ongoing interest in C–H functionalization in organic chemistry. Such technology would expedite the synthesis of high value target molecules by eliminating the pre-functionalization steps that are commonly employed in modern synthetic chemistry [1,2,3], a practice that is inherently inefficient and results in the production of large amounts of chemical waste. In particular, the use of transition metals to activate the strong C–H bond has emerged as a leading candidate to facilitate the widest range of activation/functionalization strategies while maintaining exquisite control over both reactivity and selectivity [4,5].

Interest in developing Ir(III) complexes for C–H bond functionalization has been growing rapidly [6,7]. Crabtree and coworkers showed that Cp*Ir(chelate)X (X = monodentate anionic ligand) catalyzed the selective C–H hydroxylation of alkanes and alkyl groups by NaIO_4_ [8,9,10]. Brookhart and coworkers found that (anthraphos)-Ir complexes catalyzed transfer dehydrogenation reactions of 1-hexene allowing access to *p*-xylene via tandem Diels-Alder chemistry with [11]. In related chemistry, Goldman and coworkers have disclosed the remarkable transformation of acyclic alkane precursors to substituted aromatic products [12]. In a related pincer system, Goldberg and co-workers have recently found that (phebox)Ir *n*-alkyl derivatives can be employed to generate olefin products via β-hydrogen elimination. The resulting hydride can react with O_2_ to regenerate (phebox)Ir(OAc)_2_(OH_2_) [13,14,15]. This chemistry has even been exploited in enantioselective transformations, Davies and coworkers found that (phebox)IrCl_2_(OH_2_) catalyzed asymmetric carbene C–H insertion [16].

We recently reported on the ability of an Ir(III) “pincer” complex to promote the isomerization of internal alkynes to allenes, which we proposed to occur via a C–H bond activation mechanism [17]. Allenes enjoy a unique niche as a functional group in organic chemistry. Their orthogonal cumulative π-systems provide complementary yet, in some cases, distinct reactivity compared with their alkene and alkyne cousins. Moreover, their ability to possess axial chirality sets them apart from all other functional groups. Several excellent reviews on the synthesis and utility of allenes have appeared lately [18,19,20,21]. Nonetheless, despite their increasing popularity, the development of catalytic methodologies that can access allenes (either in racemic or in enantiomerically enriched form) from readily available starting materials has dramatically lagged behind their utilization in organic synthesis.

## 2. Results and Discussion

### 2.1. Mechanistic Studies

**Scheme 1:**
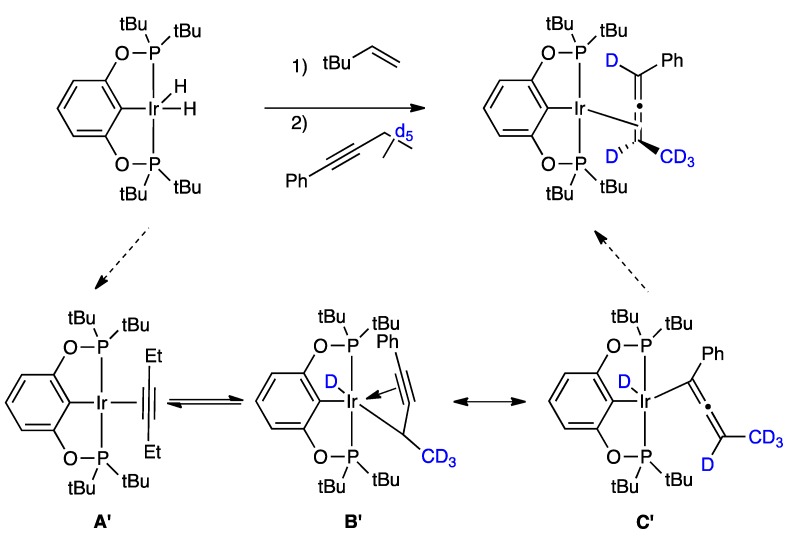
Proposed mechanism of isomerization of internal alkyne to disubstituted allene product, shown for **1a**–***d5*** → **1b**–***d5***.

We recently disclosed the ability of iridium(III) pincer complexes to effect the isomerization of internal alkynes to the corresponding allenes [17]. We proposed the transformation to occur via an initial π-bound alkyne complex (A’), observed by NMR spectroscopy, which underwent a subsequent C–H bond activation at the propargylic position (Scheme 1). The possibility of generating an Ir(III) alkyl hydride intermediate (B’) stabilized by intramolecular π-donation from the alkyne is supported, anecdotally, by the structurally similar complex [Os(η^3^-PhC_3_CHPh)(PMe_3_)_4_]^+^ [22], which has been crystallographically characterized. In our mechanistic proposal, B’ can be redrawn (via resonance) as the allenyl ligand (C’), which can undergo facile reductive elimination to afford the η^2^-allene complex product [17]. The mechanism of isomerization of internal alkynes to allenes utilizing transition metal complexes is virtually unstudied. The strong base-induced isomerization of internal alkynes to allenes is commonly known [18,19,20,21] and transition metals are known to participate in such isomerizations [23]. Alkyne to allene isomerizations within the defined transition metal complexes are rather scarce, and in some cases require acid-basic promotion [24,25,26]. Instances of the thermally induced isomerization of an internal alkyne to an allene are limited to four reports, our own iridium system [17], and examples involving Ti [27], Re [28] and Os [29] complexes. In contrast, the isomerization of alkenes by transition metals is not only known, but is an extremely valuable and intensely studied transformation [30,31,32,33,34,35,36,37]. Thus, we considered it instructive to look at the mechanisms involved in metal-catalyzed alkene isomerization, which is functionally analogous to this transformation (a 1,3-migration of hydrogen).

**Scheme 2:**
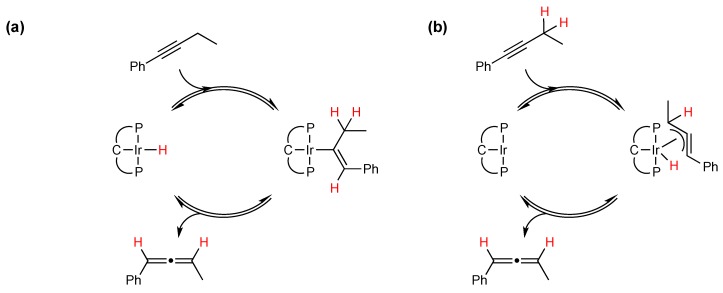
Two different classes of mechanism possible for alkyne isomerization of 1-phenylbutyne, (**a**) metal hydride insertion-elimination and (**b**) π-allyl mechanism through η^3^-allyl hydride mechanism.

Metal-catalyzed olefin isomerization is proposed to occur via one of two mechanistic pathways, insertion-elimination of a metal-hydride or the π-allyl mechanism; the analogous mechanisms for internal alkyne isomerization are shown in Scheme 2a,b, respectively. Under our reaction conditions it is unlikely the insertion-elimination mechanism is operative: (a) we do not have a metal-hydride nor is there evidence (by NMR) that one is generated *in-situ*; and (b) extensive mechanistic studies have shown that pincer-iridium complexes isomerize olefins via the π-allyl mechanism [30,31,32,33,34,35,36,37]. Our investigations turned to the use of isotopically labeled substrates as a means of probing the mechanistic pathway.

**Figure 1:**
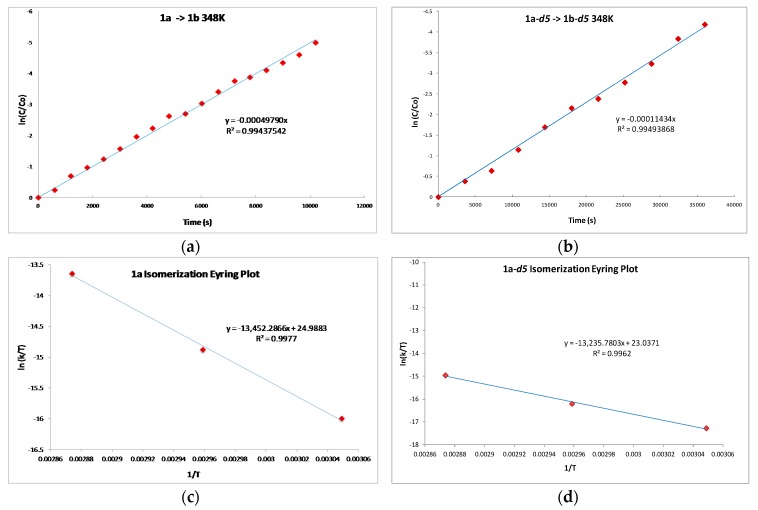
(**a**) Plot of rate of consumption of **1a** at 348 K; (**b**) Plot of rate of consumption of **1a**–***d5*** at 348 K; (**c**) The Eyring Plot for conversion of **1a** to **1b**; (**d**) The Eyring Plot for conversion of **1a**–***d5*** to **1b**–***d5***.

If C–H bond activation at the propargylic carbon is the rate-determining step (or occurs prior to the rate-determining step) then a pronounced primary kinetic isotope effect (KIE) should be observed upon moving to a substrate which incorporates deuterium at the propargylic site. Thus, we prepared the corresponding *d5*-isotopomer of **1a** [38]. As anticipated, the rate of isomerization of **1a**–***d5*** to the allene complex **1b**–***d5*** is slower than the corresponding protio-substrate **1a**; showing only ~50% conversion after 120 min compared to ~50% conversion after only 20 min for experiments conducted at 348 K. The primary KIEs were measured at three temperatures, 328, 338 and 348 K (Figure 1a,b) and exhibited values of 3.61, 3.77 and 3.75 (Table 1), respectively. Thus, the results of our isotopic labeling studies are fully consistent with our proposed mechanism.

**Table 1:** Kinetic Isotope Effect for Isomerization of **1a** → **1b** at 328 K, 338 K and 348 K.

k_H_/k_D_	*T* (K)
3.61	328.0
3.77	338.0
3.75	348.0

The activation parameters for the formation of **1b** and **1b**–***d5*** were calculated by measuring the observed rate constants for the isomerization reactions over a 20 K temperature range. The rate of conversion is conveniently determined by monitoring the ^31^P{^1^H}-NMR spectrum. The disappearance of π-bound alkyne complex (10 µmol) in a C_6_D_6_ solution (0.6 mL) was measured at 328, 338 and 348 K over at least three half-lives to allow accurate determination of rate constants (See Supplementary Materials for rate data and calculations). Employing the observed rate constants over this temperature range allowed us to construct Eyring plots (Figure 1c,d, for **1a** → **1b** and **1a**–***d5*** → **1b**–***d5***, respectively), which afforded activation entropy values (∆S‡) of 2.44 and −1.43 e.u. for the formation of **1b** and **1b**–***d5***, respectively. Similarly, the activation enthalpy (∆H‡) of formation of **1b** and **1b**–***d5*** could be determined using these Eyring plots; values of 27.05 and 26.62 kcal/mol were obtained for the conversion of **1a** → **1b** and **1a**–***d5*** → **1b**–***d5***, respectively. The near zero ∆S‡ implicates a unimolecular transition state structure for the rate-limiting event, which is supported by experiments in which the amount of added alkyne appears to have no impact on the observed rate constant.

In olefin isomerization reactions, the π-allyl mechanism results in exclusive 1,3-migration of a deuterium atom whereas deuterium incorporation into the 2-position can be observed in the insertion-elimination mechanism—arising from the reversibility of migratory insertion of the olefin into the metal-hydride bond [30,31,32,33,34,35,36,37]. Unfortunately, in neither mechanistic proposal (Scheme 2) a hydrogen (or deuterium) is placed at the 2-position and thus such an experiment cannot rule out either pathway. More conclusive evidence for the operation of a π-allyl mechanism can be garnered from crossover experiments [30,31,32,33,34,35,36,37,39,40]. Insertion mechanisms for isomerization involve the addition of a hydride derived from one olefin molecule to the double bond of a second olefin molecule; *i.e.*, the mechanism is intermolecular in contrast to the π-allyl mechanism in which a hydride effectively undergoes an intramolecular 1,3-shift. With this in mind, we treated complex 1-H_2_ with sacrificial olefin acceptor followed by equimolar amounts of **1a** and **1a–*d5***. If an iridium hydride/deuteride species is present in the system and “isomerization” (degenerate 1,2 shift of the double bond) proceeded through a hydride addition mechanism, then intermolecular H/D scrambling would be observed. Examination of the reaction mixture using NMR analysis (see Supplementary Materials for spectra) showed no such isotopic scrambling occurred.

### 2.2. Substrate Scope

Our initial report [17] described the isomerization of internal alkynes with two *n-*alkyl substituents. Given the intense interest in the chemistry of allenes and in the corresponding synthesis of a wide variety of allene substitution patterns, we undertook experiments to demonstrate the broad utility of our approach. We accomplished this by exploring a range of non-symmetrical alkynes (Table 2) incorporating aryl, and substituted-aryl substituents. Our reaction protocol is tolerant of a variety of substitution patterns, and quantitative conversion of internal alkynes to iridium-bound allene complexes is observed in all cases. Of special significance are the incorporation of electron-deficient substituents (Table 2, Entry 3) and substituents that allow further derivation of the allene moiety via well-established cross-coupling methodology (Table 2, Entry 4). The tolerance of a variety of substrates is encouraging for the development of a general and widely applicable protocol for the catalytic conversion of alkynes to allenes (*vide infra*). The relative rates of isomerization, and activation parameters, for all substrates examined in this study are qualitatively similar to those described above for substrate **1a** (see Supporting Materials).

**Table 2:** Selected Substrates for Iridium Mediated Alkyne Isomerization Reactions ^a^.

Entry	Alkyne (a)	Complex (b)
1	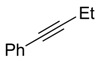	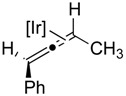
1-*d5*	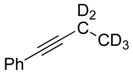	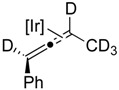
2	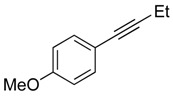	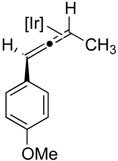
3	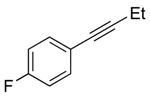	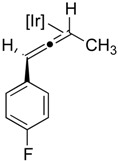
4	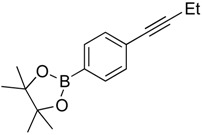	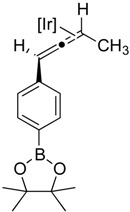

^a^ Conditions: 1:1 [Ir]:[alkyne] in C_6_D_6_, 55–75 °C, 1–60 h.

### 2.3. X-ray Structure

**Figure 2:**
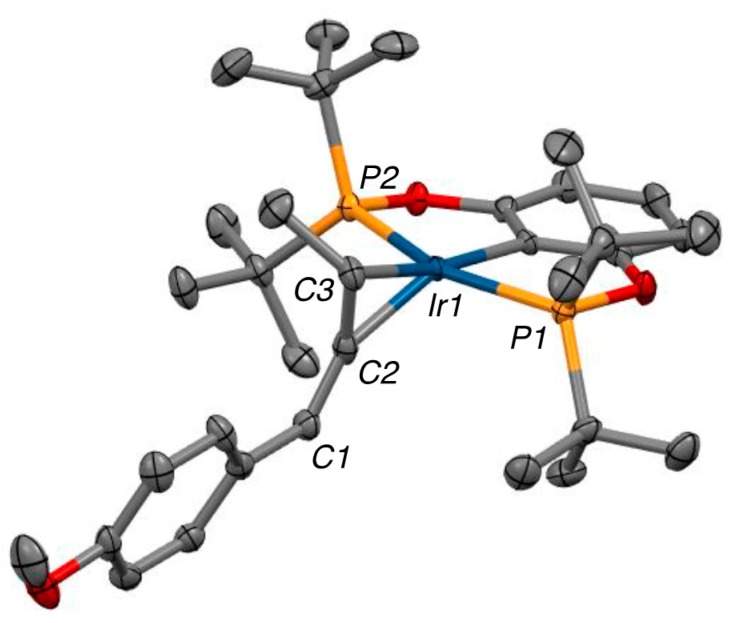
ORTEP diagram of **2b** with 40% probability thermal ellipsoids. Key bond lengths (Å) and bond angles (deg): C1–C2 = 1.324 (7), C2–C3 = 1.398 (7), Ir-C2 = 2.052 (5), Ir-C3 = 2.218 (4), C1–C2–C3 = 145.0 (5), P1-Ir-P2 = 157.01 (4).

In the solid state, the 1-(4-methoxyphenyl)-buta-1,2-diene ligand of **2b** is bound unsymmetrically to iridium through the methyl-substituted C2=C3 π-bond with a shorter Ir-C2 and a longer Ir-C3 interaction (Δd = 0.166 Å). Complex **2b** (see Figure 2) adopts a distorted trigonal-bipyramidal conformation with a P-Ir-P pincer bite angle of 157° presumably due to steric interactions emanating from the bulky *tert*-butyl phosphine substituents. Within the allene ligand, the coordinated C2=C3 bond is elongated by 0.07 Å relative to the uncomplexed C1=C2 bond, and the allene unit is bent with a C1–C2–C3 angle of 145°. This unsymmetrical binding motif and allene distortion is in keeping with our preliminary publication [17] as well as other previously published metal-allene complexes [41,42,43,44,45,46,47].

**Figure 3:**
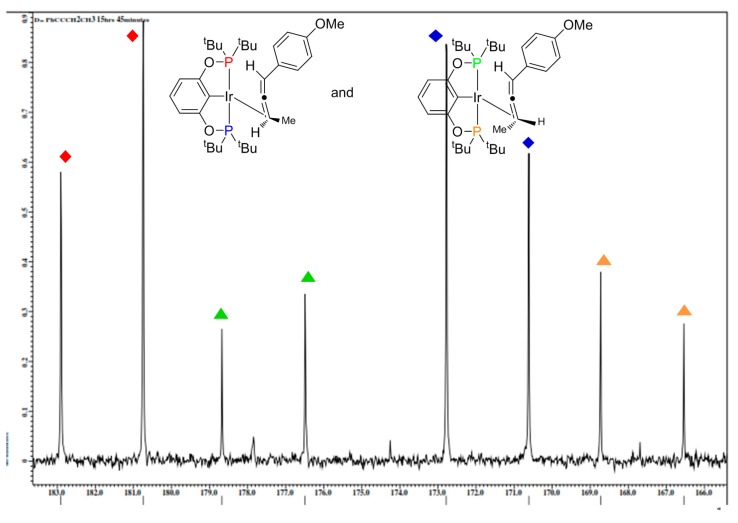

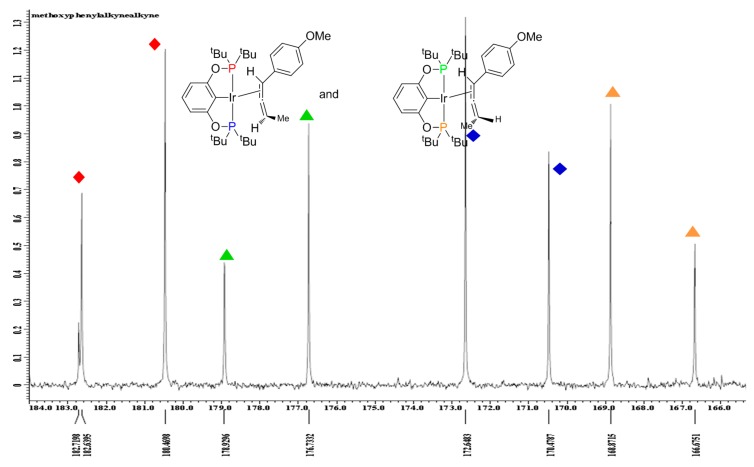
Partial ^31^P{^1^H}-NMR spectrum for **2b** showing two diastereomers in solution.

The non-symmetrical binding displayed in the solid-state is retained in the solution state, as revealed by ^31^P-MR studies (Figure 3). In the ^31^P{^1^H} spectrum of **2b** two species are observed in ~1:1 ratio. Both species display strong ^31^P-^31^P coupling of inequivalent phosphorus nuclei. Based upon side to side inequivalent phosphorous nuclei. Thus, two diastereomers are formed via binding of the planar chiral allene ligand to the pincer-iridium framework.

### 2.4. Liberation of Free Allenes Using Carbon Monoxide, Possibility of a Catalytic Isomerization Methodology

The results described above, although interesting from both fundamental and mechanistic perspectives, are stoichiometric in iridium and thus are unlikely to be widely used in synthesis. To be truly useful a catalytic variant of our methodology must be developed. When sub-stoichiometric amounts of iridium complex are employed no catalysis is observed under our standard reaction conditions. This indicated the allene in the complex is too tightly bound to the metal center and that the allene is not dissociating from the iridium atom, which would allow subsequent binding of another alkyne, closing the catalytic cycle. Examination of the molecular structure of the allene complexes (see Figure 2) reveals a sterically congested metal center, which would block further coordination of substrate to the iridium atom. This realization led us to hypothesize that a small donor ligand may be able to bind to the metal center and associatively displace the allene. We chose carbon monoxide (CO) as our ligand of choice due to the strength of metal carbonyl interactions and the small, linear, nature of the ligand.

**Scheme 3:**
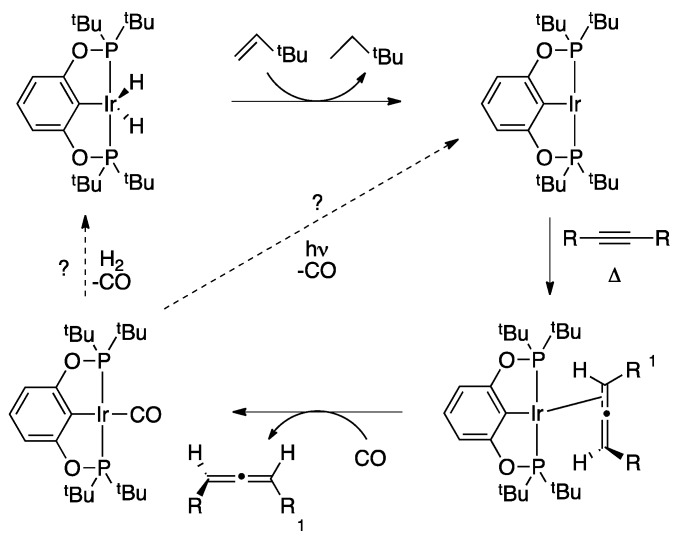
Possible strategies to render the stoichiometric alkyne → allene conversion catalytic. Promising results have been obtained with a CO promoted liberation of the allene from the iridium center.

Gratifyingly, when toluene solutions of **2b** were exposed to 1 atmosphere of CO the ^31^P{^1^H} signal corresponding to the allene complex disappeared and was replaced with the tell-tale resonance of the POCOP-Ir(CO) complex at 198.94 ppm [48]. Although we are at a very preliminary stage in our investigations, we believe this result may be the key to achieving catalytic turnover in this system as it is well-established that metal-CO interactions are susceptible to both temperature and photolysis to liberate CO and afford a coordination vacancy at the metal center (Scheme 3).

## 3. Experimental Section

### 3.1. General Information

Chemicals and solvents were purchased from commercial suppliers and were used as received except as follows: TBE and 1-Phenyl-1-butyne were degassed using freeze-pump-thaw methodology. Reactions were carried out under argon gas in a J-Young NMR tube. Chemical shifts are given in ppm. Proton and carbon NMR spectra were recorded on a Jeol 400 MHz spectrometer with Me_4_Si or solvent resonance as the internal standard. Unless otherwise noted, the NMR spectra were recorded in C_6_D_6_. Coupling constants (*J*) are given in Hertz (Hz). The terms m, s, d, t, q, quint., sext., vt, represent multiplet, singlet, doublet, triplet, quartet, quintet, sextet, virtual triplet respectively. The term br means that the signal is broad. X-ray diffraction experiments were performed at the Chemistry Department X-ray Diffraction Facility of the Texas Tech University using a Bruker Smart Apex II CCD diffractometer. All data were collected at low temperature using graphite-monochromated Mo-Kα radiation. Gas chromatography-mass spectrometry (GC-MS) was performed on an electron ionization time-of-flight (EI-TOF) mass spectrometer.

Crystallographic data for the structure of **2b** have been deposited with the Cambridge Crystallographic Data Centre (deposition number: CCDC 1435142). CCDC-1435142 contain the supplementary crystallographic data for this paper. These data can be obtained free of charge from the Cambridge Crystallographic Data Center, 12 union Road, Cambridge CB2 1EZ, UK; fax: (+44) 1223-336-033; e-mail: deposit @ccdc.cam.ac.uk.

### 3.2. Synthesis

*Synthesis of* **1a**–**d5**: To a THF (30 mL) solution of phenylacetylene (2.00 g, 19.6 mmol) was added *n*-butyllithium (1.6 M, 13.5 mL, 21.6 mmol) at −40 °C. After stirring at this temperature for 30 min, HMPA (1 mL, 5.5 mmol) was added and the reaction mixture was allowed to warm to room temperature over the course of ~40 min. Subsequently, bromoethane-*d5* (2.46 g, 21.6 mmol) was added to the solution and the mixture was heated at reflux temperature for 24 h. The reaction was then quenched with a saturated aqueous NH_4_Cl solution. The aqueous solution was extracted with ether (3 × 1 mL), and the organic phase dried over MgSO_4_, and evaporated to dryness. The residues were eluted through a silica gel column (hexane: ethyl acetate) to afford analytically pure **1a**–***d5*** (1.9 g, 75%) [14]. ^2^D-NMR (400 MHz, CD_2_Cl_2_): 1.14 (s, 2D, ≡*–*C*D_2_*–), −0.036 (s, 3D, ≡*–*C*D_2_*–C*D_3_*).

*General Procedure for the Synthesis of Non-symmetrical Alkynes (Described for* **2a***):* To a THF (10 mL) solution of 1-ethynyl-4-methoxybenzene (0.50 g, 3.8 mmol) was added *n*-butyllithium (1.6 M, 2.6 mL, 4.2 mmol) at −40 °C. After stirring at this temperature for 30 min, HMPA (0.17 mL, 1.1 mmol) was added and the reaction allowed to warm to room temperature over the course of ~40 min. Subsequently, iodoethane (0.66 g, 4.2 mmol) was added to the solution and the mixture was heated at reflux temperature for 24 h. The reaction was then quenched with a saturated aqueous NH_4_Cl solution. The aqueous solution was extracted with ether (3 × 10 mL), and the organic phase dried over MgSO_4_, and evaporated to dryness. The residues were eluted through a silica gel column to afford **2a** as an analytically pure white solid. The NMR analysis agreed with the reported values [40,49].

*Typical Procedure for Preparation of η^2^-Allene Complexes* **1b**–**4b**: In a J-Young NMR tube, (POCOP)IrH_2_ (12 mg, 20 µmol) was dissolved in C_6_D_6_ (*ca*. 0.5 mL) and *ca.* 1.5 eq. *tert*-butylethylene added via microsyringe. The removal of H_2_ with concomitant formation of 1 eq. of *tert*-butylethane is easily monitored using both ^1^H- and ^31^P{^1^H}-NMR spectroscopy. Addition of 1 eq. of alkyne affords exclusively the η^2^-adducts of alkynes **1a**–**4a**. The solutions of η^2^-alkyne complexes, prepared as above, were warmed to *ca.* 75 °C for 5–10 h to reach full conversion to the allene complexes **1b**–**4b**. Definitive assignment of diastereomers is hampered by closely overlapping peaks in the ^1^H-NMR spectra and tentative assignments are made on the basis of previously reported NMR data of related allene complexes and 2-D NMR experiments.

*Procedure for the Isolation of Allenes using CO (described for complex* **2b***):* In a J-Young NMR tube, (POCOP)IrH_2_ (12 mg, 20 µmol) was dissolved in C_6_D_6_ (*ca.* 0.5 mL) and *ca.* 1.5 eq. *tert*-butylethylene added via microsyringe. The removal of H_2_ with concomitant formation of 1 eq. of *tert*-butylethane is easily monitored using both ^1^H- and ^31^P{^1^H}-NMR spectroscopy. Addition of 1 eq. of alkyne affords exclusively the η^2^-adduct of alkyne **2a**. The solution of η^2^-alkyne complex, prepared as above, was warmed to *ca.* 75 °C for 12 h to reach full conversion to the Ir-η^2^-**2b** allene complex. After cooling to room temperature, the J-Young NMR tube was degassed using freeze-pump-thaw techniques. Subsequently, CO gas (1 atm.) was admitted to the tube for ~1 min. The reaction solution within the J-Young NMR tube was mixed for 10 min and the tube transferred to the NMR. *In-situ* analysis shows generation of the known (POCOP)Ir(CO) complex and liberation of the allene from the metal center.

## 4. Conclusions

The pincer iridium complex [(POCOP)Ir] is capable of the facile isomerization of internal alkynes into disubstituted allenes. Deuterium labeling experiments provided support for a mechanism in which the metal-promoted activation of a C–H bond plays a key role and a primary kinetic isotope effect was measured. The rates of isomerization of a variety of alkyne substrates were measured over a broad temperature range, which allowed the thermodynamic parameters to be determined. Finally, exposure of the η^2^-allene complexes to CO atmosphere allowed isolation of the free allenes providing a potential catalytic pathway to the conversion of internal alkynes to allenes.

## Supplementary Materials

Supplementary materials can be accessed at: http://www.mdpi.com/1420-3049/20/11/19686/s1.
