# Supplementary material for: Isomerization of Internal Alkynes to Iridium(III) Allene Complexes via C–H Bond Activation: Expanded Substrate Scope, and Progress towards a Catalytic Methodology
Source: Molecules. 2015 Nov 10;20(11):20195–205. doi: 10.3390/molecules201119686 (PMC6332053; doi:10.3390/molecules201119686)
Supplement: Supplementary file 1 [file molecules-20-19686-s001.pdf]

Supplementary Materials: Isomerization of Internal Alkynes to Iridium(III) Allene Complexes via C–H Bond Activation: Expanded Substrate Scope, and Progress towards a Catalytic Methodology

Neha Phadke and Michael Findlater

Table of Contents

General remarks	S2
Typical procedure for in-situ preparation of η^2–allene complexes	S2
Spectroscopic Characterization of **1b**	S2
Spectroscopic Characterization of **1b–d5**	S3
Spectroscopic Characterization of **2b**	S3
Spectroscopic Characterization of **3b**	S4
Spectroscopic Characterization of **4b**	S5
Crossover Experiments	S6
CO Liberation of allene from iridium complex	S7
Kinetics of allene complex formation	S9
Plot of rate of consumption of **1a** at 328 K trial 1	S10
Plot of rate of consumption of **1a** at 328 K trial 2	S10
Plot of rate of consumption of **1a** at 338 K trial 1	S11
Plot of rate of consumption of **1a** at 338 K trial 2	S11
Plot of rate of consumption of **1a** at 348 K trial 1	S12
Plot of rate of consumption of **1a** at 348 K trial 2	S12
Eyring Plot for conversion of **1a** → **1b**	S13
Plot of rate of consumption of **1a–d5** at 328 K trial 1	S14
Plot of rate of consumption of **1a–d5** at 328 K trial 2	S14
Plot of rate of consumption of **1a–d5** at 338K trial 1	S15
Plot of rate of consumption of **1a–d5** at 338K trial 2	S15
Plot of rate of consumption of **1a–d5** at 348K trial 1	S16
Plot of rate of consumption of **1a–d5** at 348K trial 2	S16
Eyring Plot for conversion of **1a–d5** → **1b–d5**	S16
Kinetic Isotope Effect	S17
Plot of rate of consumption of **2a** at 328 K trial 1	S17
Plot of rate of consumption of **2a** at 328 K trial 2	S17
Plot of rate of consumption of **2a** at 338K trial 1	S18
Plot of rate of consumption of **2a** at 338K trial 2	S18
Plot of rate of consumption of **2a** at 348K trial 1	S19
Plot of rate of consumption of **2a** at 348K trial 2	S19
Eyring Plot for conversion of **2a** → **2b**	S20
Plot of rate of consumption of **4a** at 328 K trial 1	S20
Plot of rate of consumption of **4a** at 328 K trial 2	S21
Plot of rate of consumption of **4a** at 338K trial 1	S21
Plot of rate of consumption of **4a** at 338K trial 2	S22
Plot of rate of consumption of **4a** at 348K trial 1	S22
Plot of rate of consumption of **4a** at 348K trial 2	S23
Eyring Plot for conversion of **4a** → **4b**	S23
X-ray analysis of 2b	S24

General Remarks. Chemicals and solvents were purchased from commercial suppliers and were used as received except as follows: TBE and 1-Phenyl-1-butyne were degassed. Reactions were carried out under argon gas in a *J*-Young nmr tube. Chemical shifts are given in ppm. Unless otherwise noted, the NMR spectra were recorded in C_6D_6. Coupling constants (*J*) are given in Hertz (Hz). The terms m, s, d, t, q, quint., sext., vt, represent multiplet, singlet, doublet, triplet, quartet, quintet, sextet, virtual triplet respectively. The term br means that the signal is broad. X-ray diffraction experiments were performed at the Chemistry Department X-ray Diffraction Facility of the Texas Tech University.

Typical Procedure for Preparation of η²-allene Complexes (1b–4b): In a J-Young NMR tube, (POCOP)IrH_2 (12 mg, 20 μmol) was dissolved in C_6D_6 and ca. 1.5 eq. *tert*-butylethylene added via microsyringe. The removal of H_2 with concomitant formation of 1 eq. of *tert*-butylethane is easily monitored using both ^{1}H and ^{31}P {^{1}H} NMR spectroscopy. Addition of 1 eq. of alkyne affords exclusively the η²-adducts of alkynes **1a–5a**. The solutions of η²-alkyne complexes, prepared as above, were warmed to *ca.* 75 °C for 5–10 h to reach full conversion to the allene complex. Definitive assignment of diastereomers is hampered by closely overlapping peaks in the ^{1}H NMR spectra and tentative assignments are made on the basis of previously reported NMR data of related allene complexes and 2-D NMR experiments.

Spectroscopic Characterization of **1b**:

Trans-(POCOP)Ir(η²-(Ph)C(H)=C=C(H)(CH₃)) and Cis-(POCOP)Ir(η²-(Ph)C(H)=C=C(H)(CH₃)) (1b & 1b'): ^{1}H NMR (500 MHz, C_6D_6): δ 7.46–6.83 (m, 8H, Ar), 6.51 (br d, 1H, Ph-*H*C=C=C), 4.15 (br t, 1H, $^3J_{H\text{-}H}$ = 6.0 Hz, C=C=C*H*–CH₃), 1.96 (d, 3H, $^3J_{H\text{-}H}$ = 6.0 Hz, C=C=CH–C*H*₃), 1.36-1.05 (m, 36H, C(C*H*₃)₃). ^{31}P {^{1}H} (162 MHz, C_6D_6): δ 182.36 (AB, $J_{A\text{-}B}$ = 351.13 Hz), 171.70 (AB, $J_{A\text{-}B}$ = 351.13 Hz).

^{1}H-NMR (500 MHz, C_6D_6): δ 7.46–6.83 (m, 8H, Ar), 5.36 (br d, 1H, Ph-*H*C=C=C), 4.15 (br t, 1H, $^3J_{H\text{-}H}$ = 6.0 Hz, C=C=C*H*–CH₃), 1.36–1.05 (m, 36H, C(C*H*₃)₃), 0.79 (d, 3H, $^3J_{H\text{-}H}$ = 14 Hz, C=C=CH–C*H*₃). ^{31}P {^{1}H} (162 MHz, C_6D_6): δ 177.58 (AB, $J_{A\text{-}B}$ = 353.29 Hz), 167.6327 (AB, $J_{A\text{-}B}$ = 353.29 Hz).

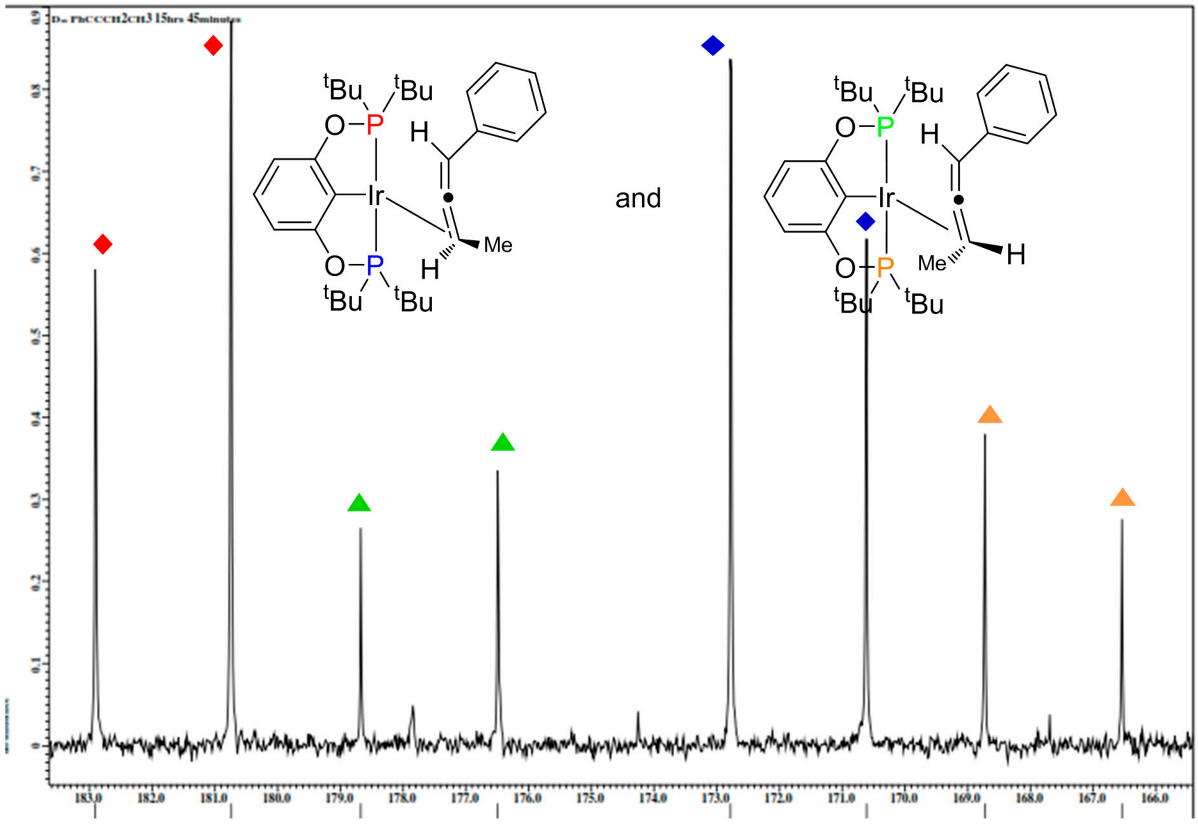

Spectroscopic Characterization of **1b–d5**:

Trans-(POCOP)Ir(η^2-(Ph)C(D)=C=C(D)(CD$_3$)) and Cis-(POCOP)Ir(η^2-(Ph)C(D)=C=C(D)(CD$_3$)) (1b–d5 & 1b–d5′): ^{31}P {^{1}H} (162 MHz, C_6D_6): δ 181.72 (AB, $J_{A\text{-}B}$ = 348.96 Hz), 171.70 (AB, $J_{A\text{-}B}$ = 348.96 Hz). ^{31}P {^{1}H} (162 MHz, C_6D_6): δ 177.48 (AB, $J_{A\text{-}B}$ = 353.29 Hz), 167.60 (AB, $J_{A\text{-}B}$ = 353.29 Hz).

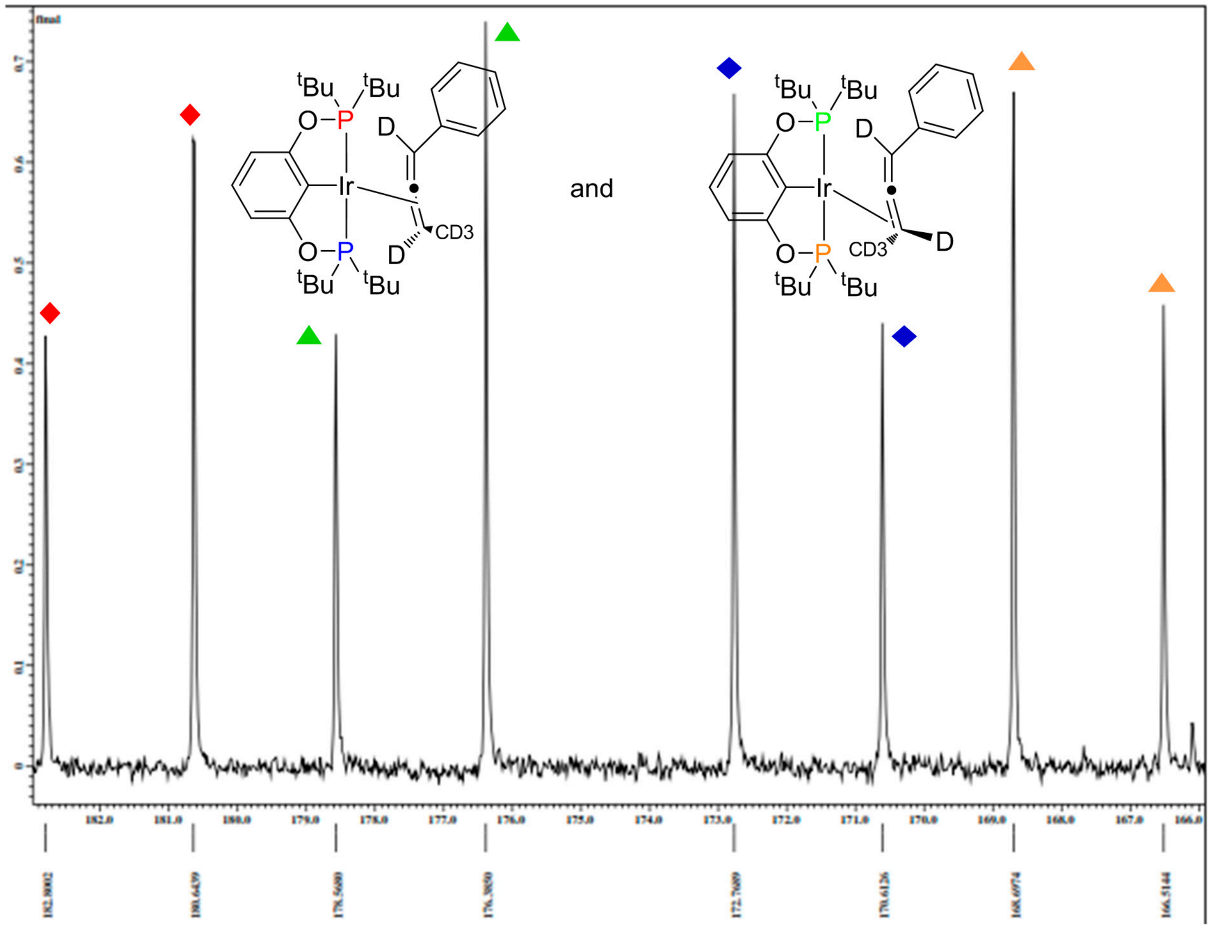

Spectroscopic Characterization of **2b**:

Trans-(POCOP)Ir(η^2-(p-MeO-Ph)C(D)=C=C(D)(CD$_3$)) and Cis-(POCOP)Ir(η^2-(p-MeO-Ph)C(D) =C=C(D)(CD$_3$)) (2b & 2b′): ^{31}P {^{1}H} (162 MHz, C_6D_6): δ 181.55 (AB, $J_{A\text{-}B}$ = 351.13 Hz), 171.56 (AB, $J_{A\text{-}B}$ = 351.13 Hz). ^{31}P {^{1}H} (162 MHz, C_6D_6): δ 178.38 (AB, $J_{A\text{-}B}$ 355.46 = 355.46 Hz), 167.77 (AB, $J_{A\text{-}B}$ = 351.13 Hz).

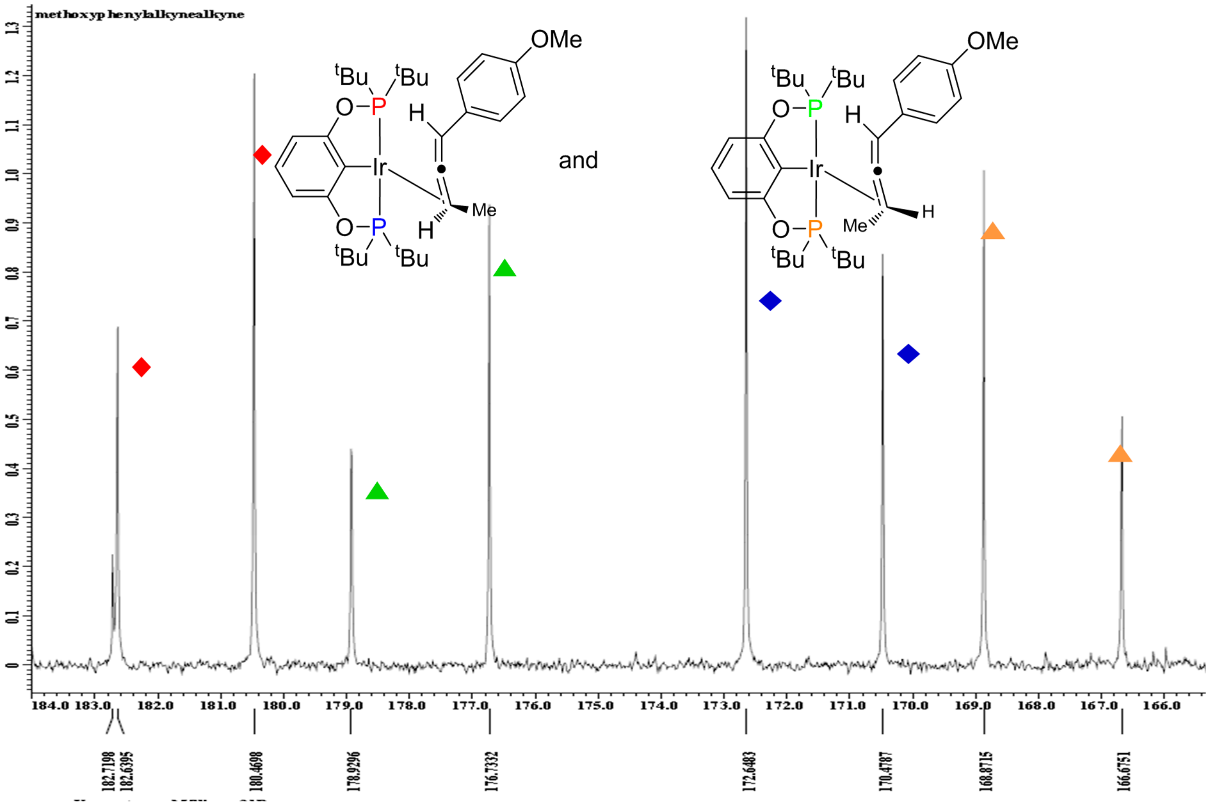

Spectroscopic Characterization of **3b**:

Trans-(POCOP)Ir(η²-(Ph)C(D)=C=C(D)(CD₃)) and Cis-(POCOP)Ir(η²-(Ph)C(D)=C=C(D)(CD₃)) (3b & 3b'): ^{31}P {^{1}H} (162 MHz, C_6D_6): δ 182.97 (AB, J_{A-B} = 348.96 Hz), 171.61 (AB, J_{A-B} = 348.96 Hz). ^{31}P {^{1}H} (162 MHz, C_6D_6): δ 172.70 (AB, J_{A-B} = 351.13 Hz), 167.72 (AB, J_{A-B} = 351.13 Hz).

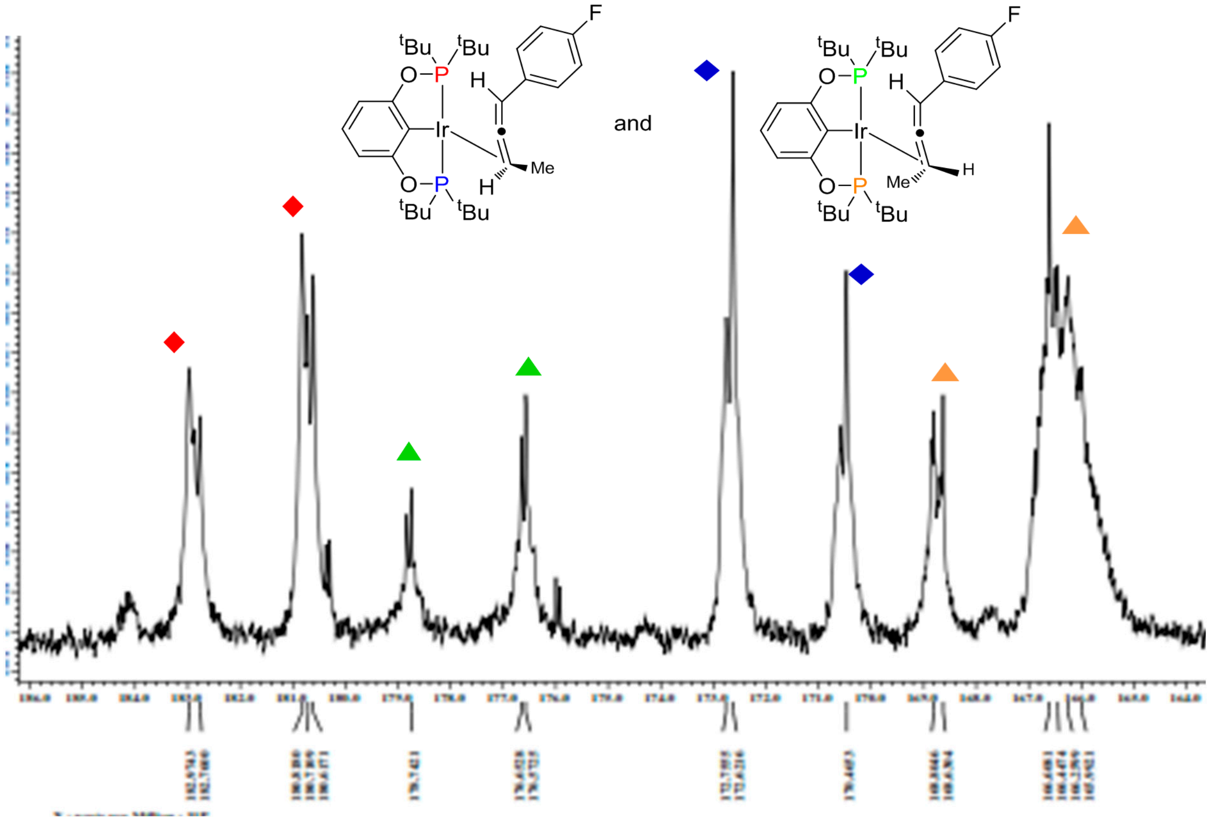

Spectroscopic Characterization of **4b**:

Trans-(POCOP)Ir(η^2-(Ph)C(D)=C=C(D)(CD_3)) and Cis-(POCOP)Ir(η^2-(Ph)C(D)=C=C(D)(CD_3)) (4b & 4b′): $^{31}P\{^1H\}$ (162 MHz, C_6D_6): δ 182.09 (AB, J_{A-B} = 346.79 Hz), 171.86 (AB, J_{A-B} = 346.79 Hz). $^{31}P\{^1H\}$ (162 MHz, C_6D_6): δ 177.56 (AB, J_{A-B} = 353.29 Hz), 167.62 (AB, J_{A-B} = 353.29 Hz).

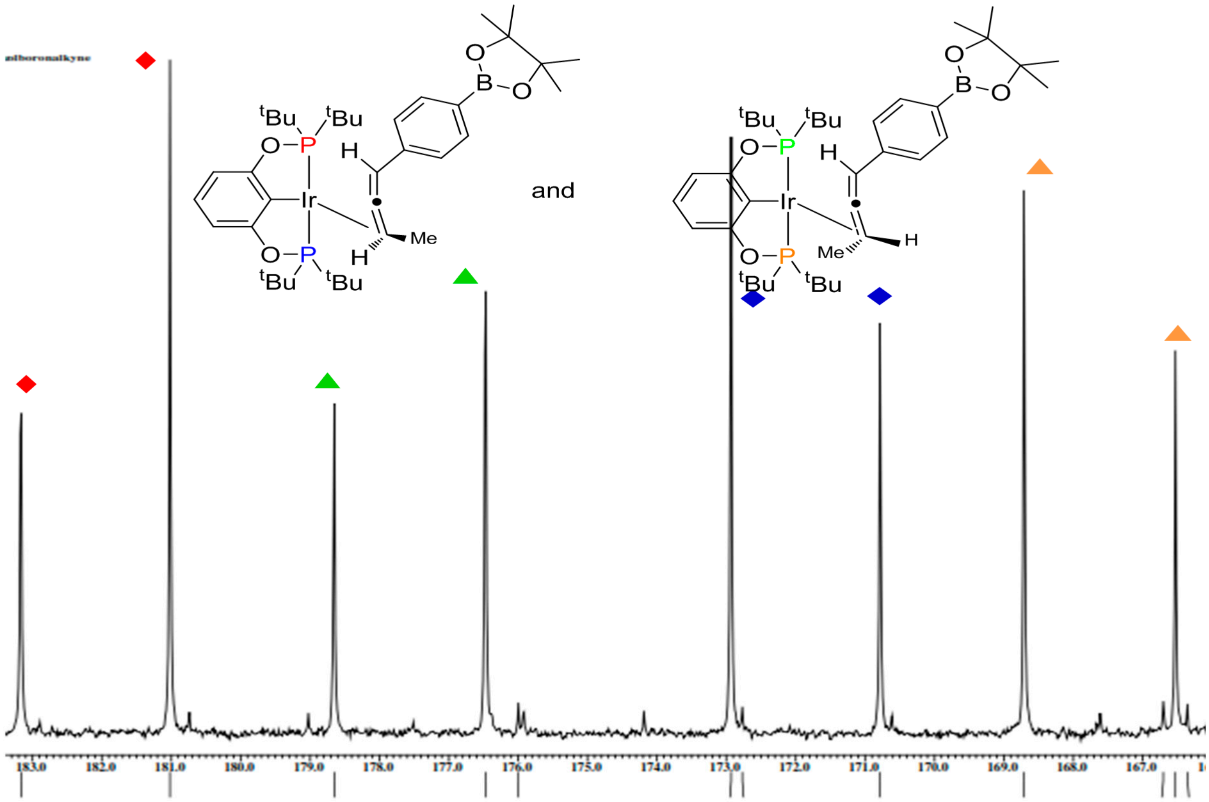

Crossover Experiment

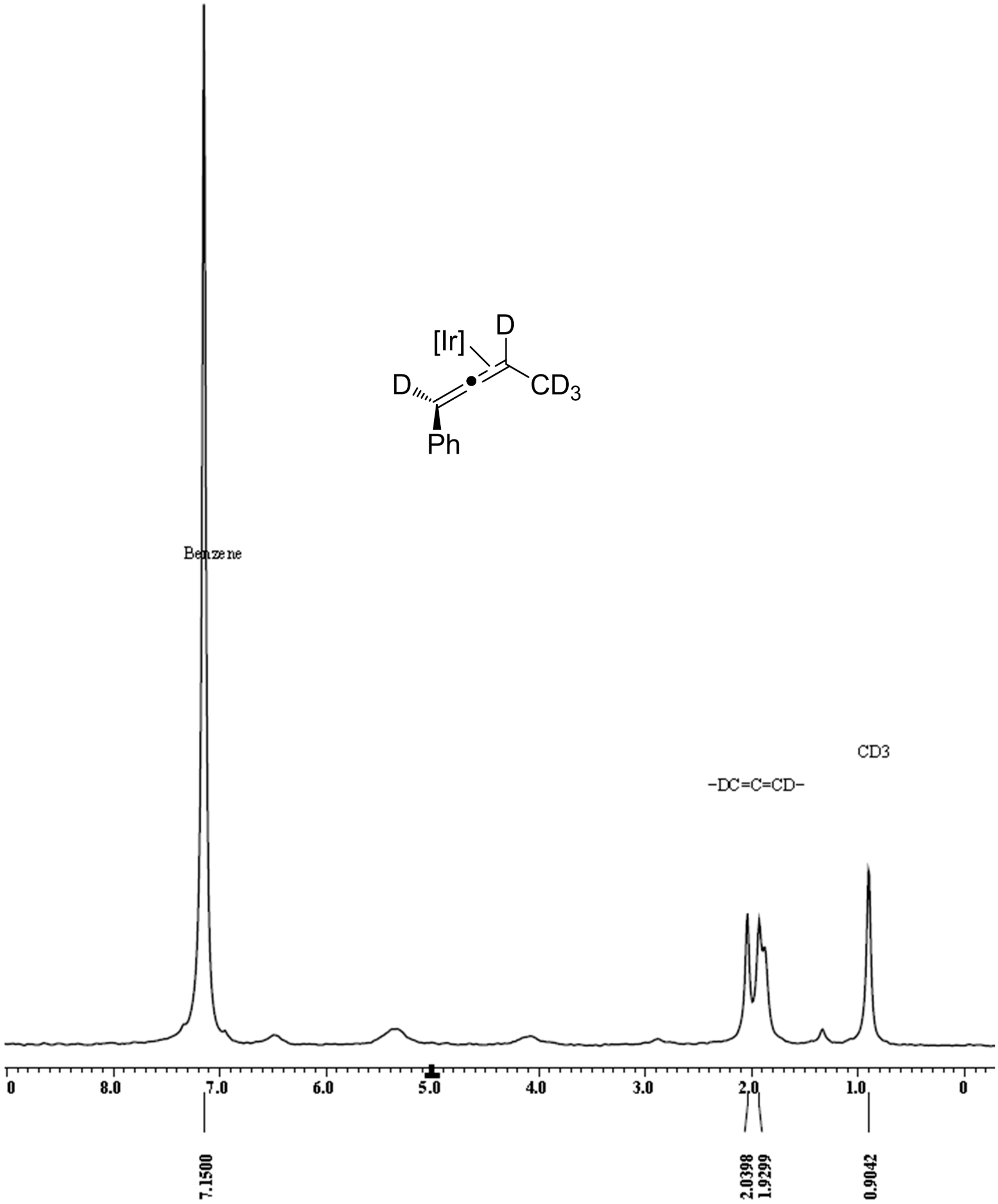

Figure S1. [2]D NMR of crossover labeling experiment of the mixture of **1b** and **1b–*d5*** allene.

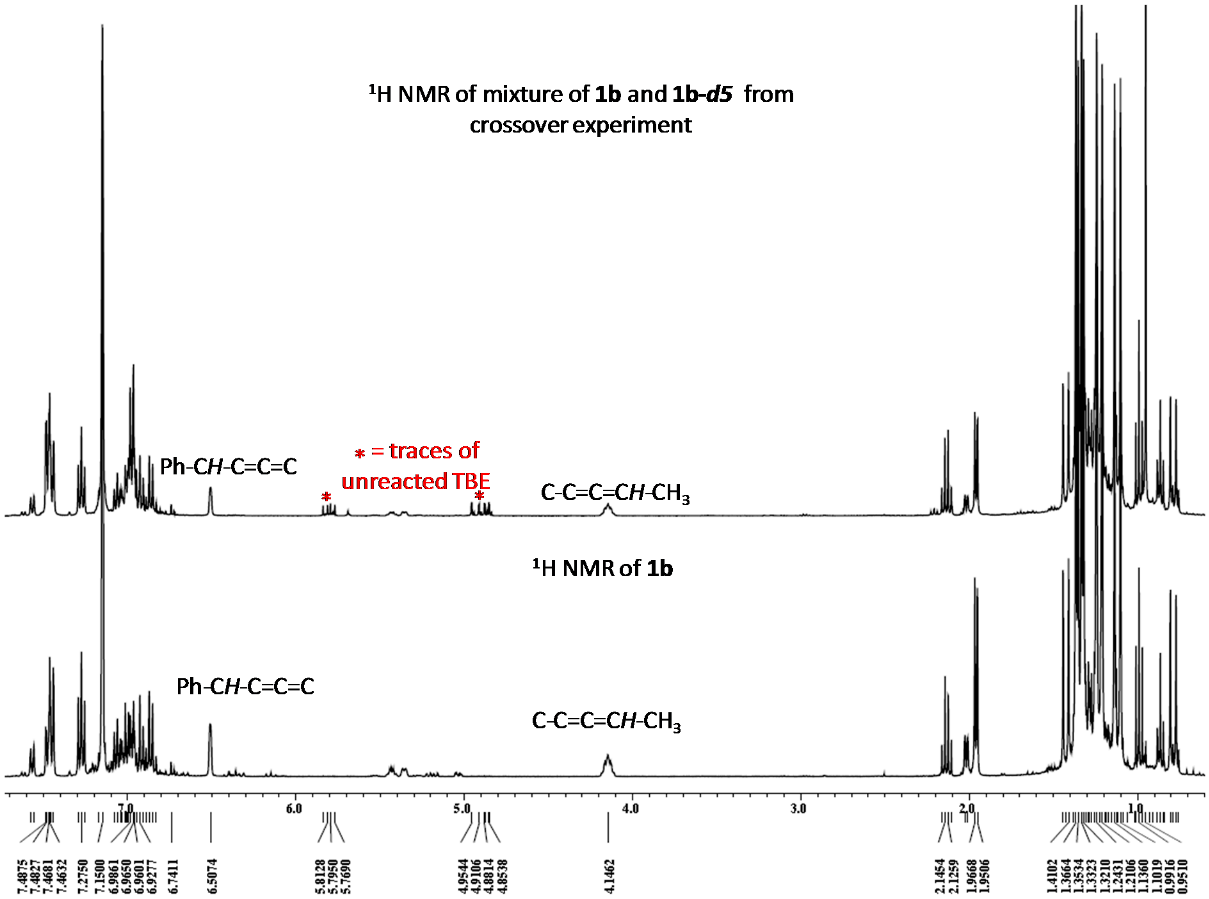

Figure S2. Stacked plot of product **1b** and mixture of **1b** and **1b**–*d5*.

Liberation of Allene Using CO

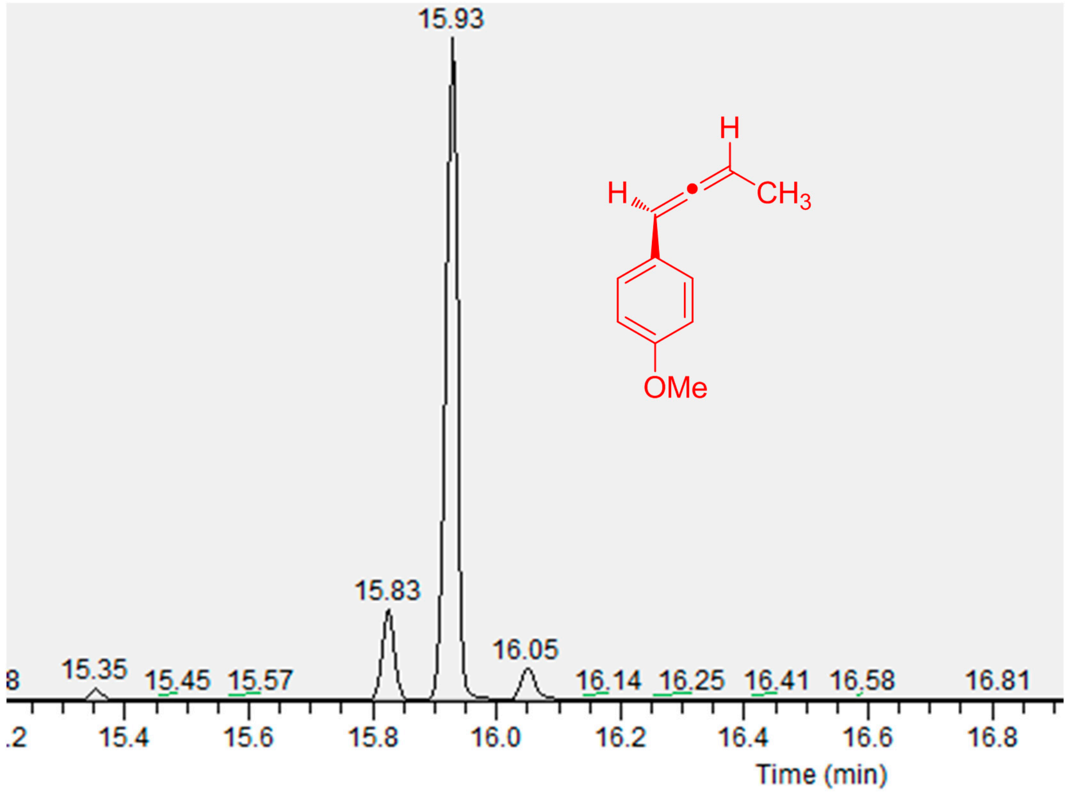

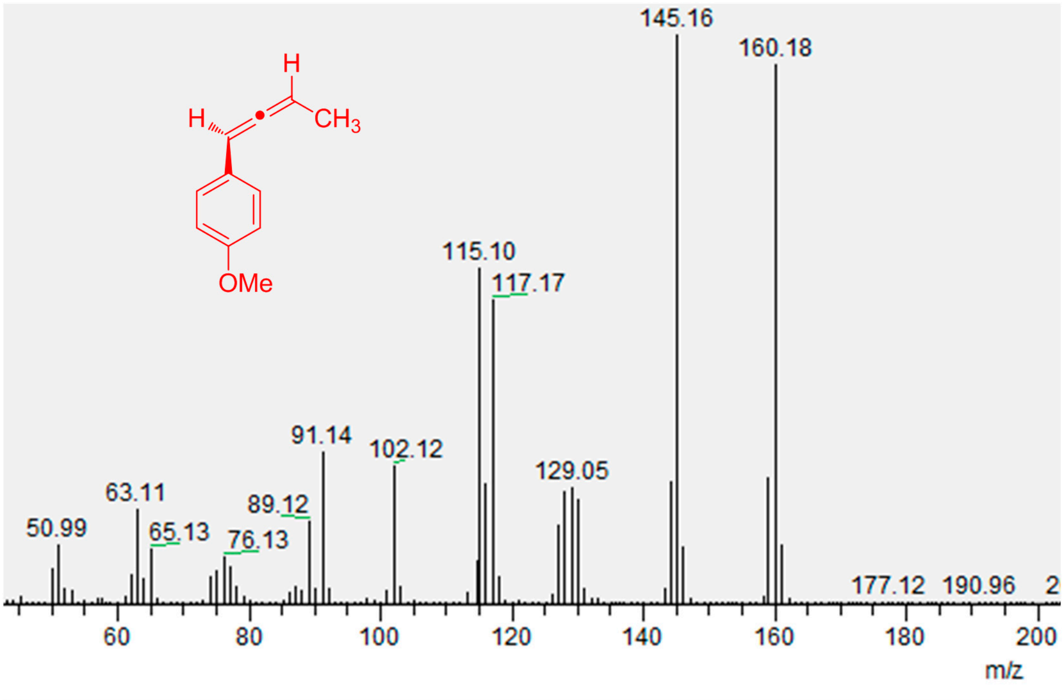

Figure S3. GC-MS of the isolated **2b** product from the CO experiment.

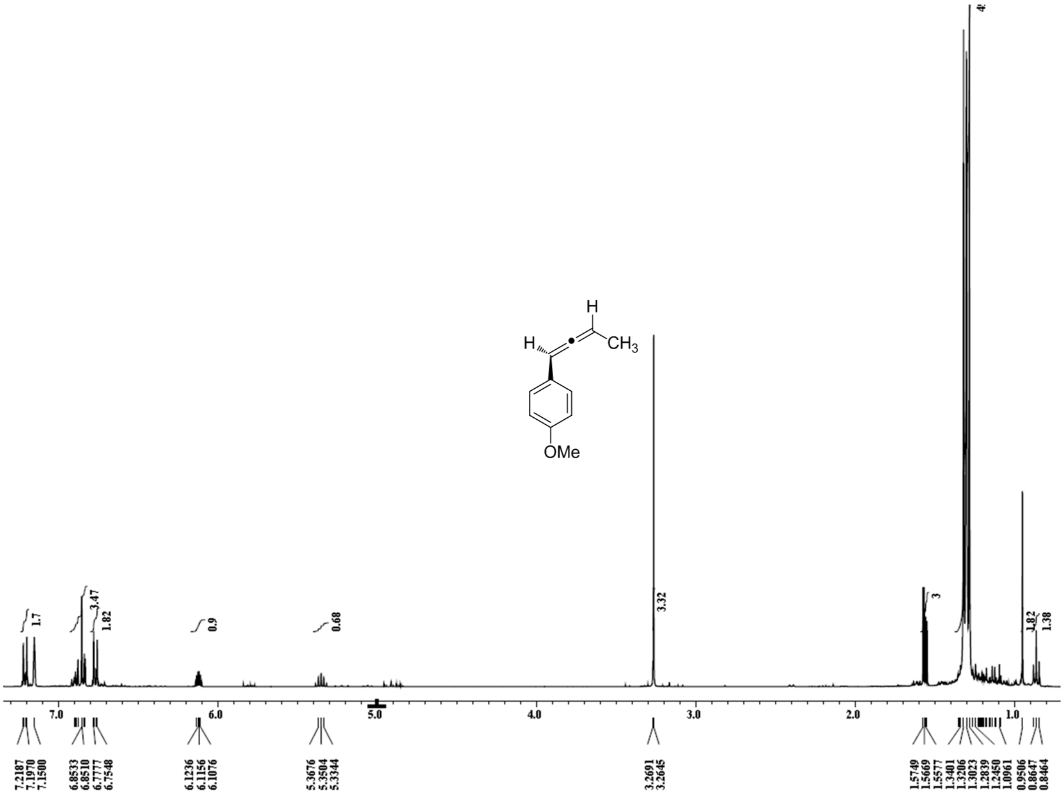

Figure S4. The ^{1}H NMR of isolated allene from **2b** after CO treatment (mixture of allene and Ir-CO complex).

^{1}H NMR: Mixture of isolated allene **2b** and the Ir-CO complex

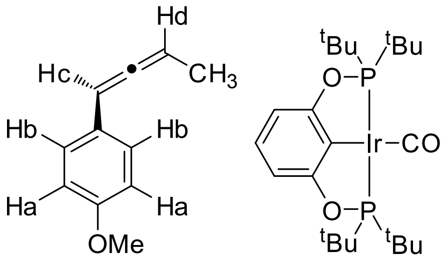

Isolated allene 2b ^{1}H NMR: 7.22-7.19 (m, 2Ha), 6.77–6.75 (m, 2Hb), 6.13–6.10 (m, Hc), 5.35 (m, Hd), 3.26 (s, $-OCH_3$), 1.57-1.55 (m, $-CH_3$).
Ir-CO complex ^{1}H NMR: 6.83 (m, Ar, 3H),1.30 (vt, 36H, 2 x P(tBu)$_2$). ^{31}P NMR: 198.9

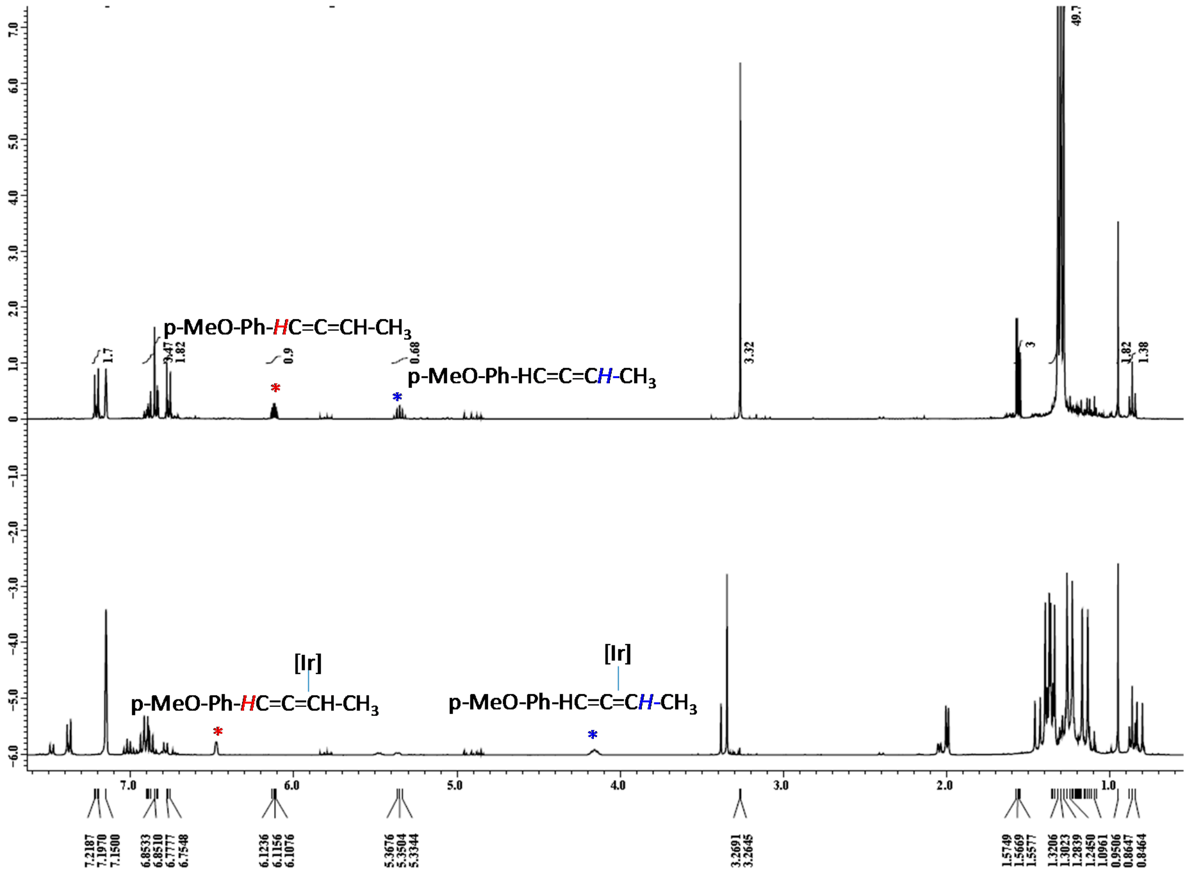

Figure S5. Stacked ^{1}H NMR of **2b** and isolated allene from **2b** after CO treatment.

NMR Kinetics of Allene Complex Formation: The rate of conversion of **1a** to **1b** was determined by monitoring the disappearance of the singlet of **1a** in the ^{31}P {^{1}H} NMR spectrum. The disappearance of **1b** (10 µmol) in a C_6D_6 solution (0.6 mL) was measured at 348, 338 and 328 K over at least 3 half-lives to allow accurate determination of rate constants. A plot of ln [**1a**$_t$]/[**1b**$_i$] vs time was essentially linear, the average (k_{obs} = 4.173 × 10^{-4} (348 K); 1.169 × 10^{-4} (338 K); 3.718 × 10^{-5} (328 K)). By plotting the change in reaction rate *vs.* temperature an Eyring Plot could be constructed (ln(k/T) *vs.* 1/T). The rates of isomerization, and activation parameters, for **1a–d5, 2a and 4a** are qualitatively similar to those of **1a**. Representative plots are given below; in addition the corresponding thermodynamic parameters are calculated.

Kinetic Plots:

Plot of rate of consumption of **1a** at 328 K, 338 K, and 348 K

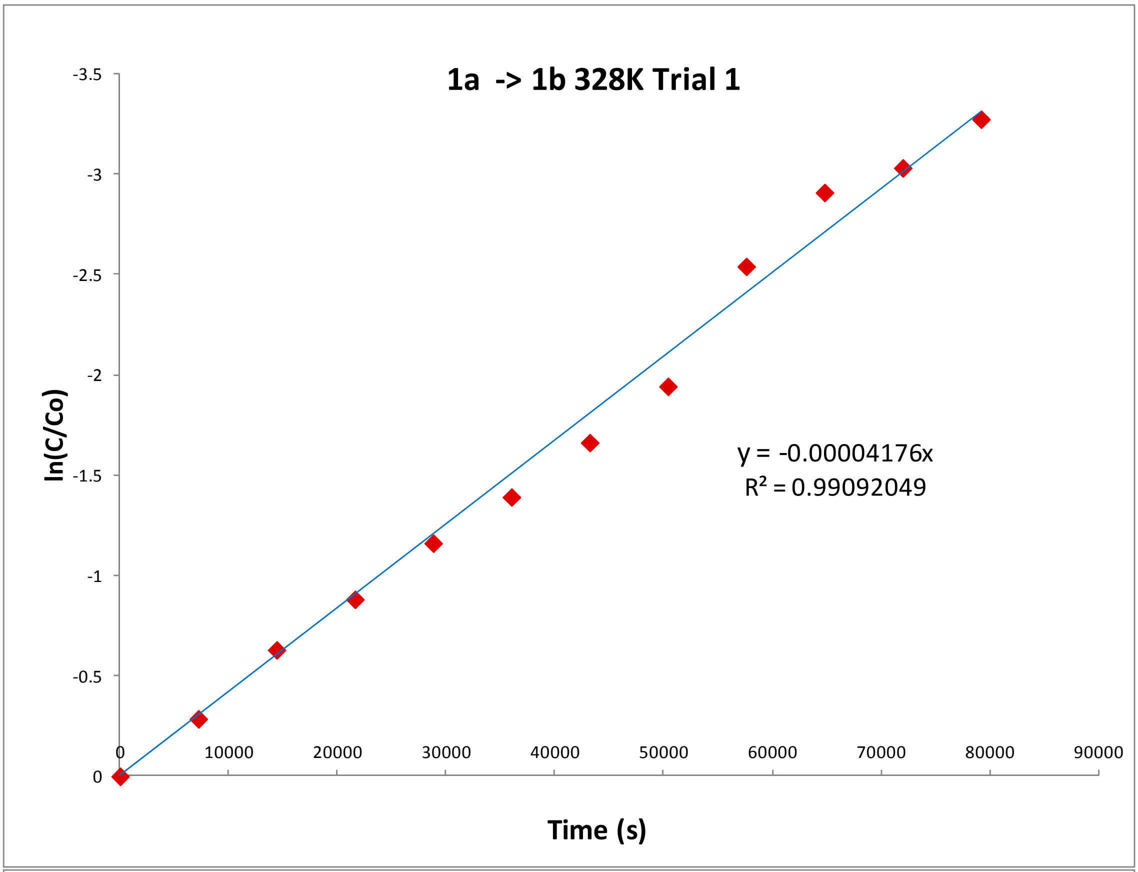

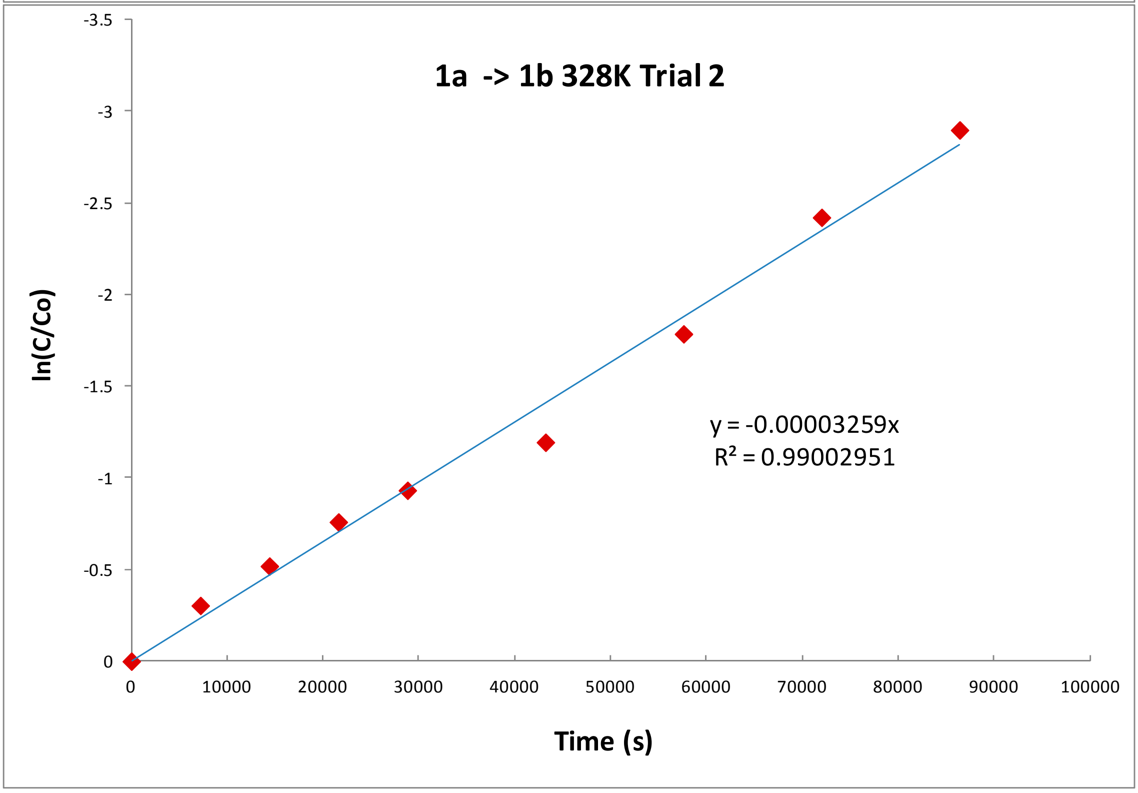

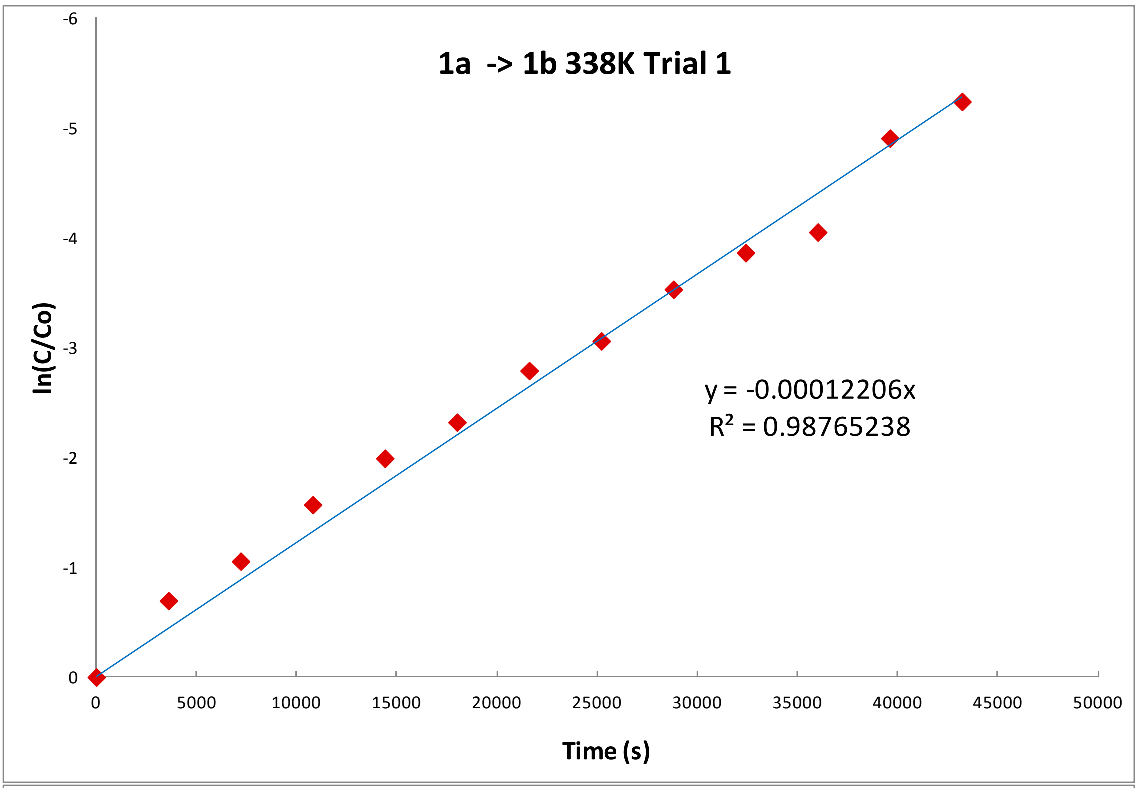
1a -> 1b 338K Trial 1
y = -0.00012206x
R² = 0.98765238
ln(C/Co)
Time (s)
-6
-5
-4
-3
-2
-1
0
0
5000
10000
15000
20000
25000
30000
35000
40000
45000
50000

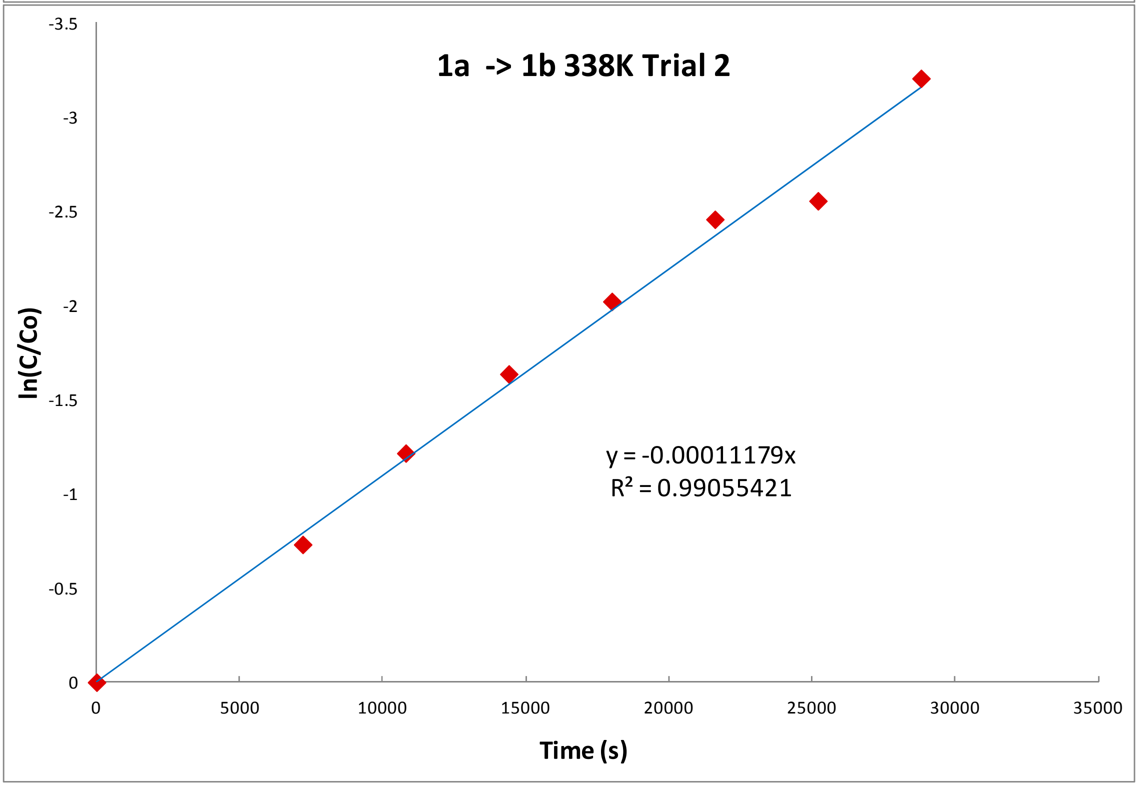
1a -> 1b 338K Trial 2
y = -0.00011179x
R² = 0.99055421
ln(C/Co)
Time (s)
-3.5
-3
-2.5
-2
-1.5
-1
-0.5
0
0
5000
10000
15000
20000
25000
30000
35000

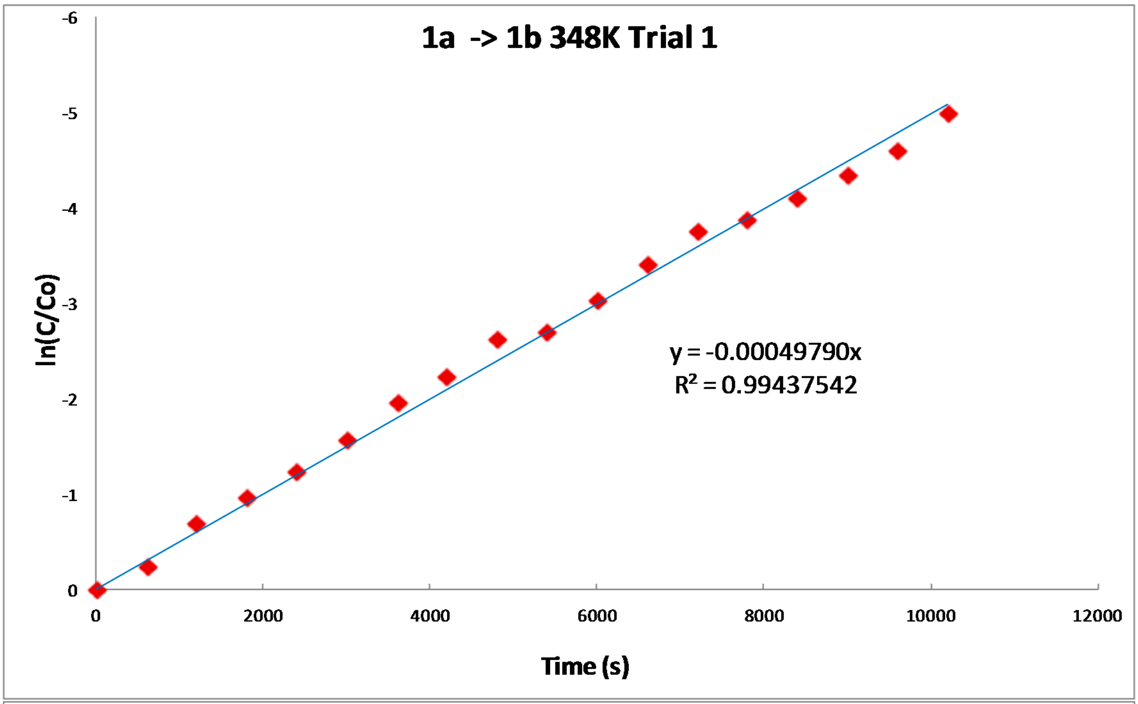
1a -> 1b 348K Trial 1
ln(C/Co)
Time (s)
y = -0.00049790x
R² = 0.99437542
-6
-5
-4
-3
-2
-1
0
0
2000
4000
6000
8000
10000
12000

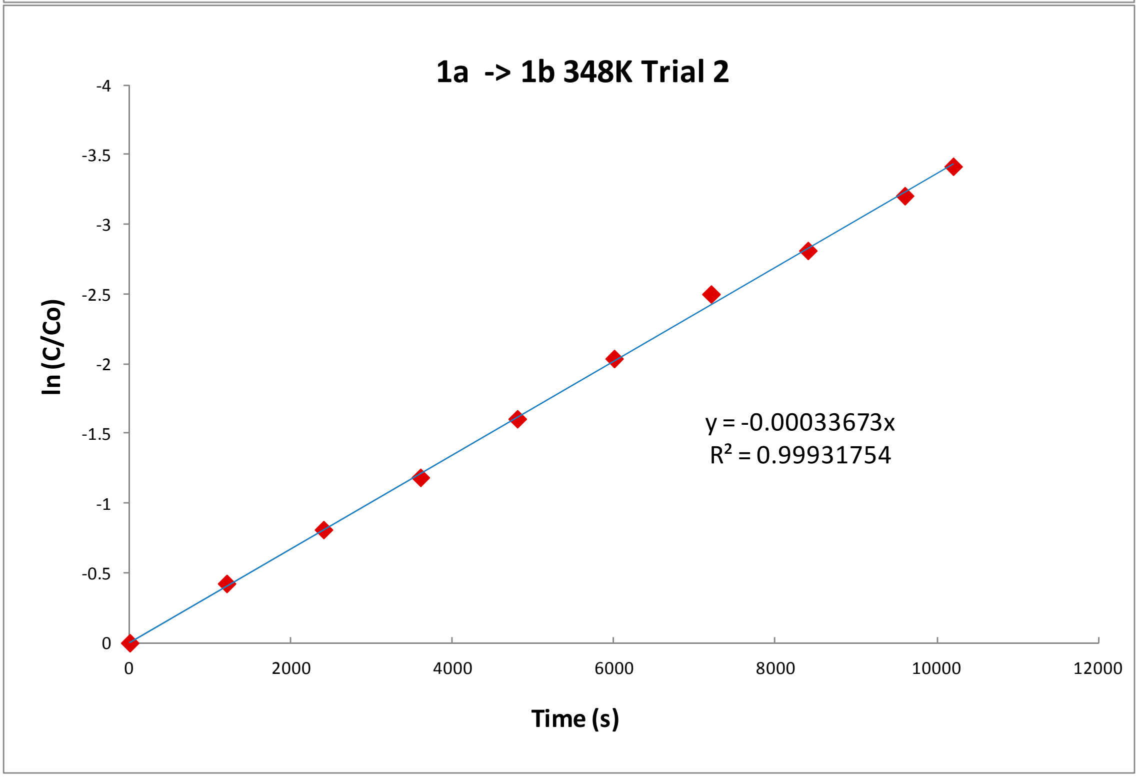
1a -> 1b 348K Trial 2
ln (C/Co)
Time (s)
y = -0.00033673x
R² = 0.99931754
-4
-3.5
-3
-2.5
-2
-1.5
-1
-0.5
0
0
2000
4000
6000
8000
10000
12000

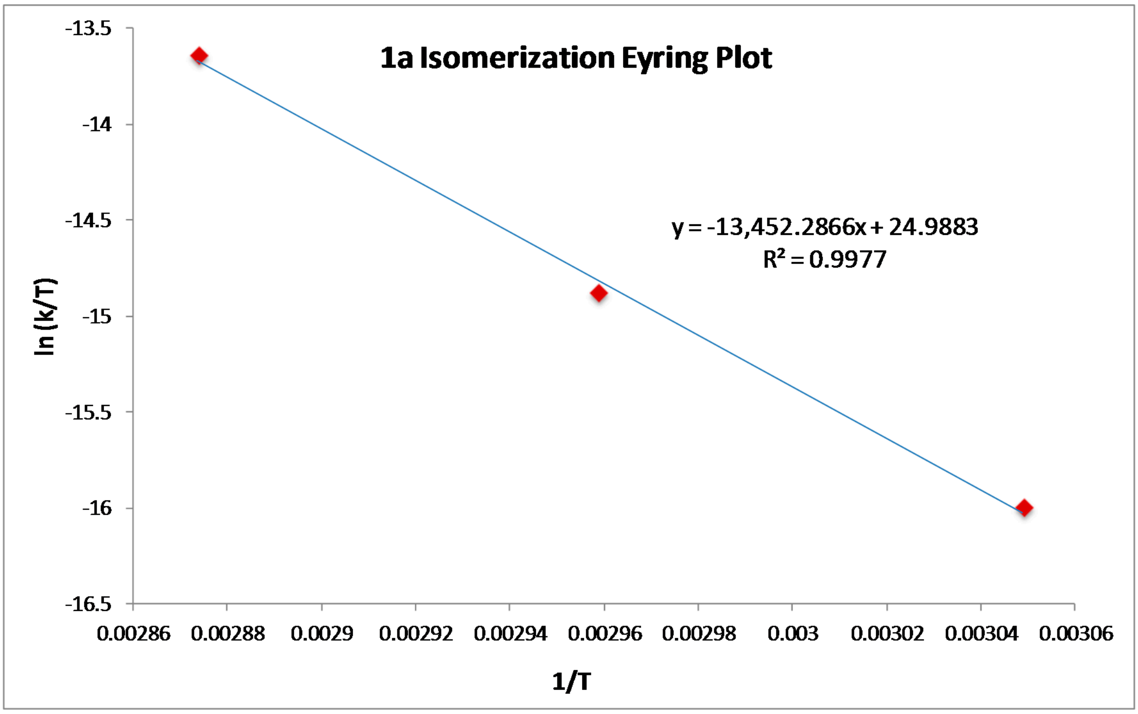

T (K)	k_H (s^{-1}) T rial 1	k_H (s^{-1}) Trial 2	k_H (s^{-1}) Average	ΔG‡ (kcal/mol) Average
328.0	4.176 × 10	3.259×10^{-5}	3.7175×10^{-5}	26.23
338.0	1.2206×10^{-4}	1.1179×10^{-4}	1.16925×10^{-4}	26.26
348.0	4.979×10^{-4}	3.3673×10^{-4}	4.17315×10^{-4}	26.18

ΔH‡ = 27.05 kcalmol^{-1}; ΔS‡ = 2.44 e.u.

Plot of rate of consumption of **1a–d5** at 328 K, 338 K, and 348 K

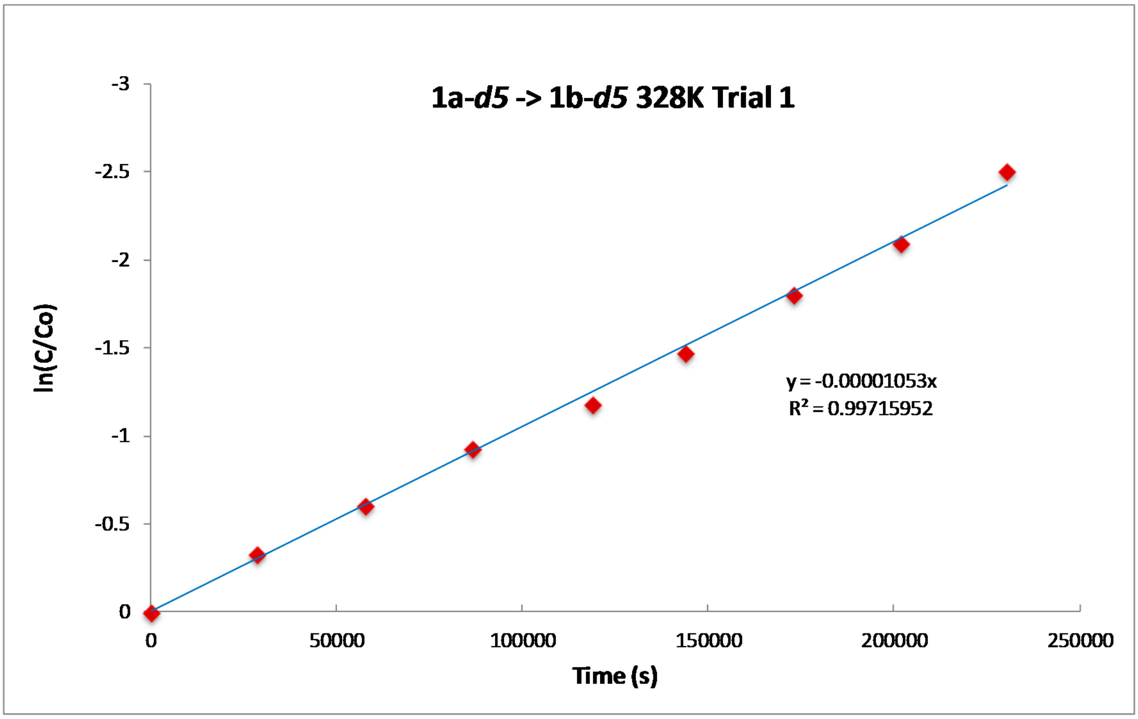

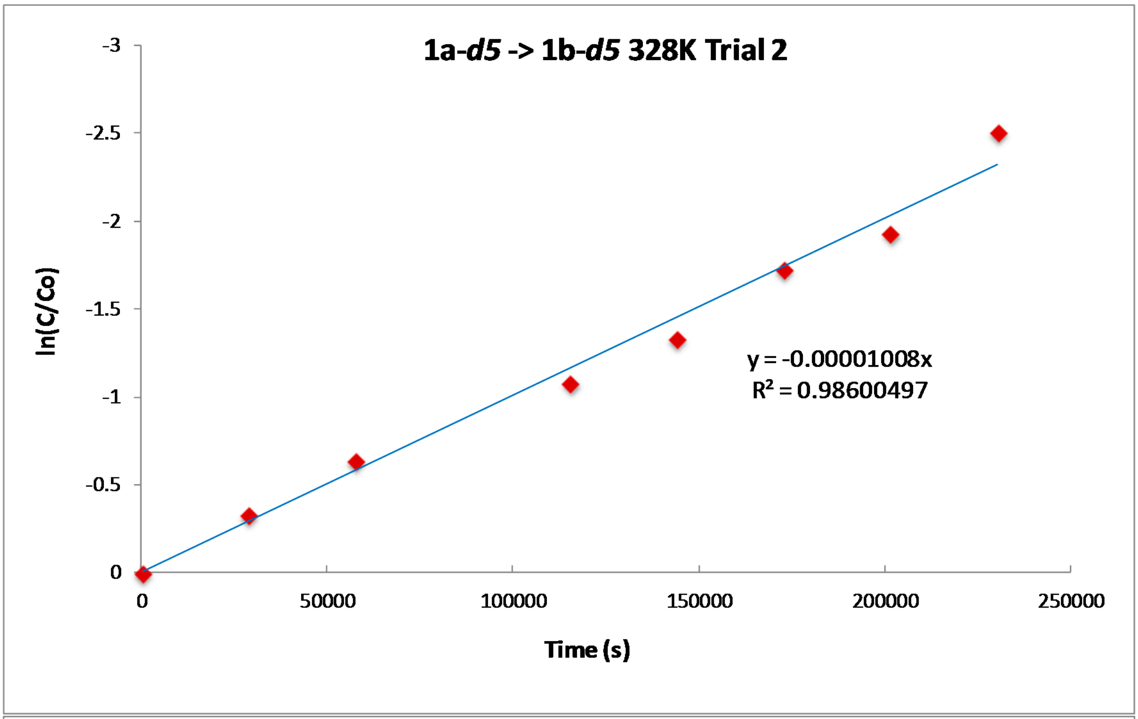
1a-*d5* -> 1b-*d5* 328K Trial 2
ln(C/Co)
-3
-2.5
-2
-1.5
-1
-0.5
0
0
50000
100000
150000
200000
250000
Time (s)
y = -0.00001008x
R² = 0.98600497

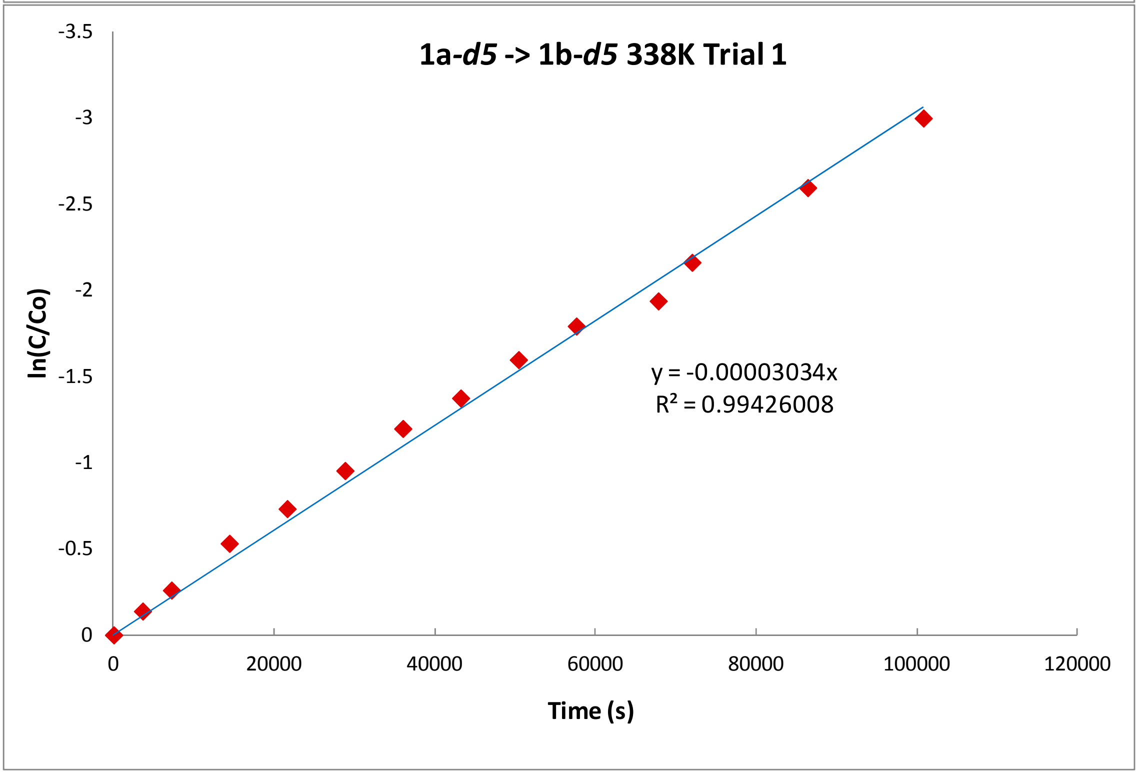
1a-*d5* -> 1b-*d5* 338K Trial 1
ln(C/Co)
-3.5
-3
-2.5
-2
-1.5
-1
-0.5
0
0
20000
40000
60000
80000
100000
120000
Time (s)
y = -0.00003034x
R² = 0.99426008

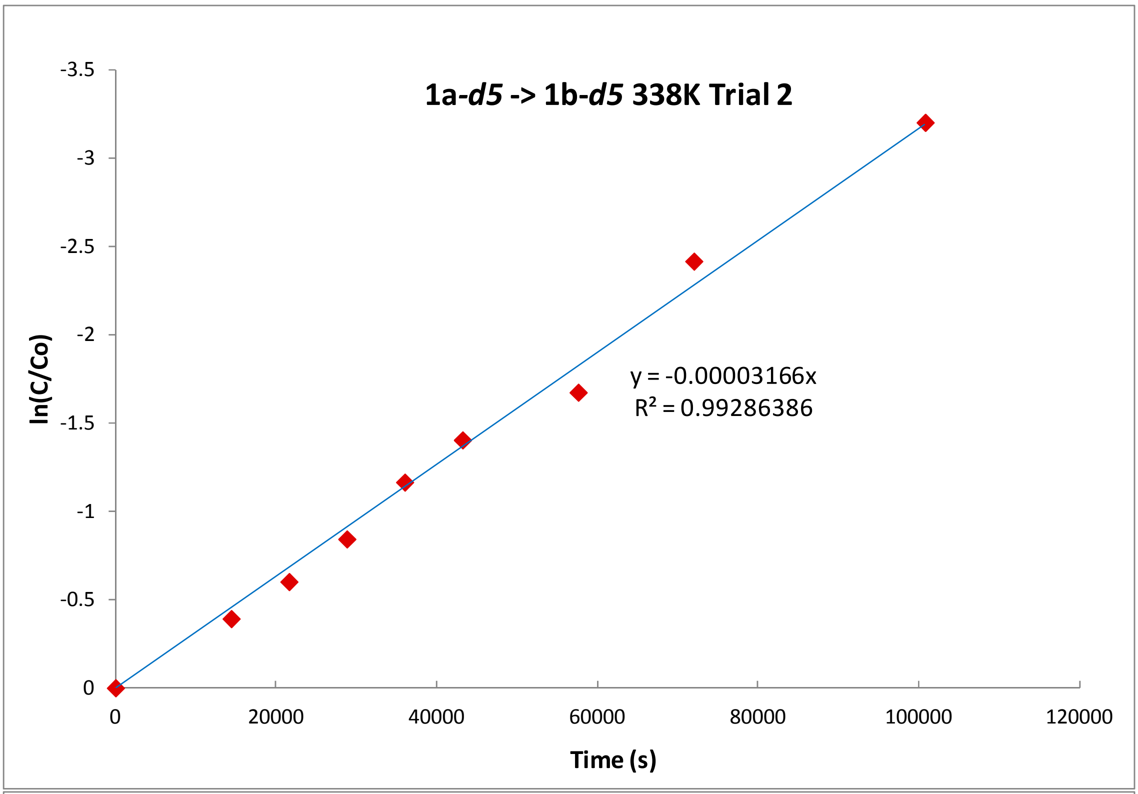
1a-d5 -> 1b-d5 338K Trial 2
y = -0.00003166x
R² = 0.99286386
ln(C/Co)
Time (s)
-3.5
-3
-2.5
-2
-1.5
-1
-0.5
0
0
20000
40000
60000
80000
100000
120000

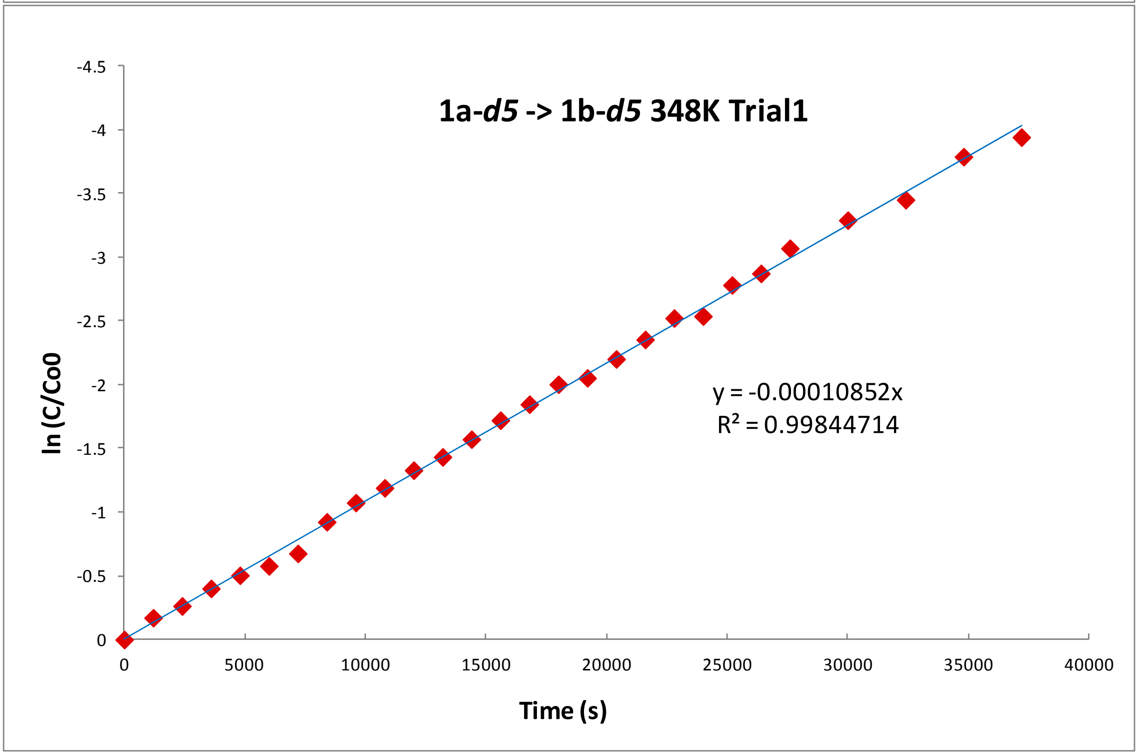
1a-d5 -> 1b-d5 348K Trial1
y = -0.00010852x
R² = 0.99844714
ln (C/Co0
Time (s)
-4.5
-4
-3.5
-3
-2.5
-2
-1.5
-1
-0.5
0
0
5000
10000
15000
20000
25000
30000
35000
40000

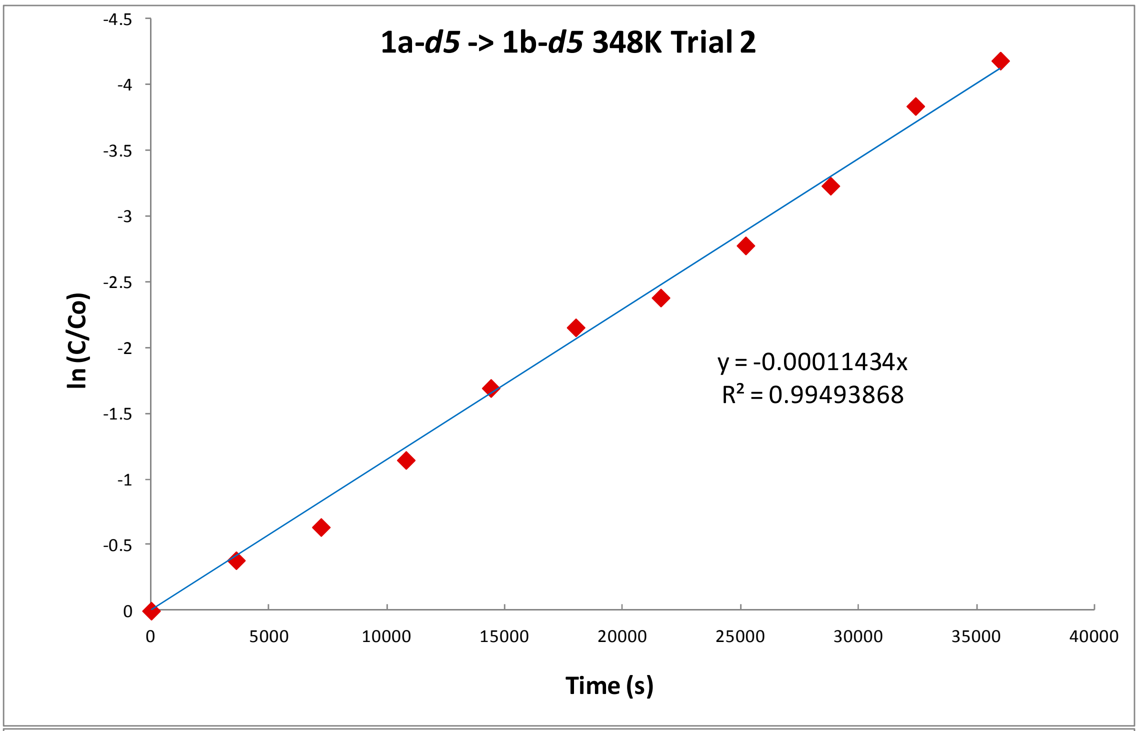

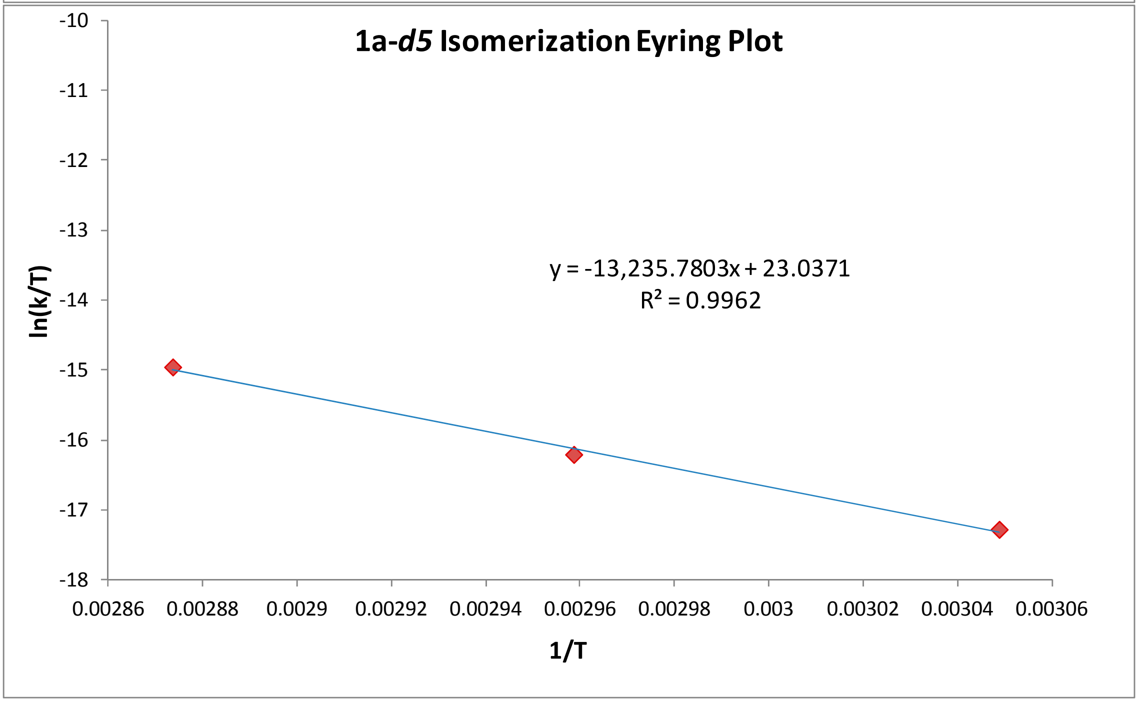

T (K)	k_D (s^{-1}) Trial 1	k_D (s^{-1}) Trial 2	k_D (s^{-1}) Average	ΔG‡ (kcal/mol) Average
328.0	1.053×10^{-5}	1.008×10^{-5}	1.031×10^{-5}	27.07
338.0	3.034×10^{-5}	3.166×10^{-5}	3.100×10^{-5}	27.17
348.0	1.0852×10^{-4}	1.1434×10^{-4}	1.1143×10^{-4}	27.09

Δ H‡ = 26.62 kcalmol^{-1}; ΔS‡ = −1.43 e.u.

Kinetic Isotope Effect:

Average k_H/k_D	T (K)
3.61	328.0
3.77	338.0
3.75	348.0

Plot of rate of consumption of **2a** at 328, 338, 348 K respectively.

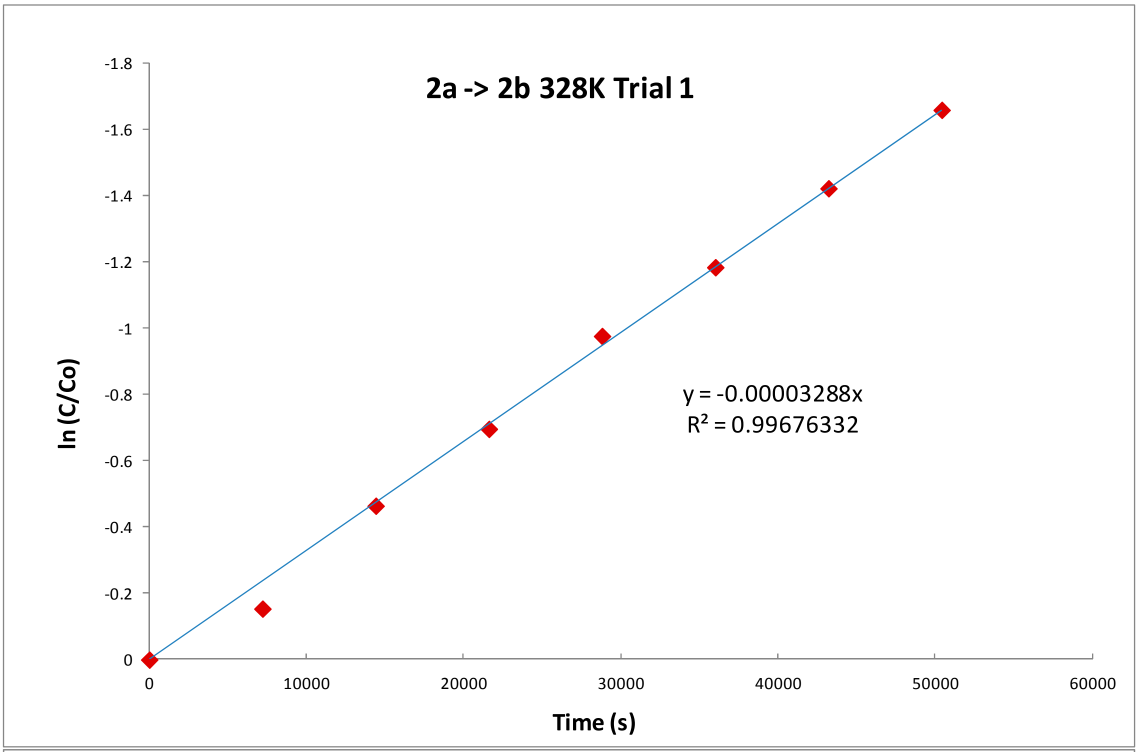

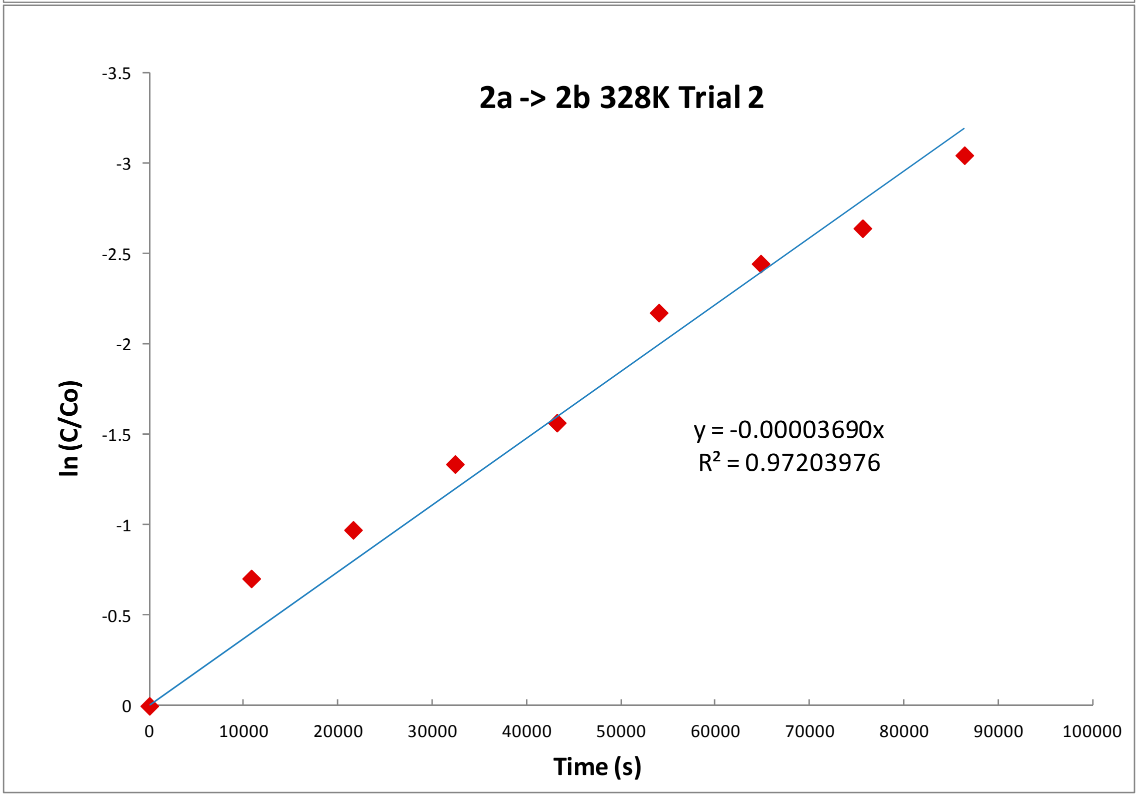

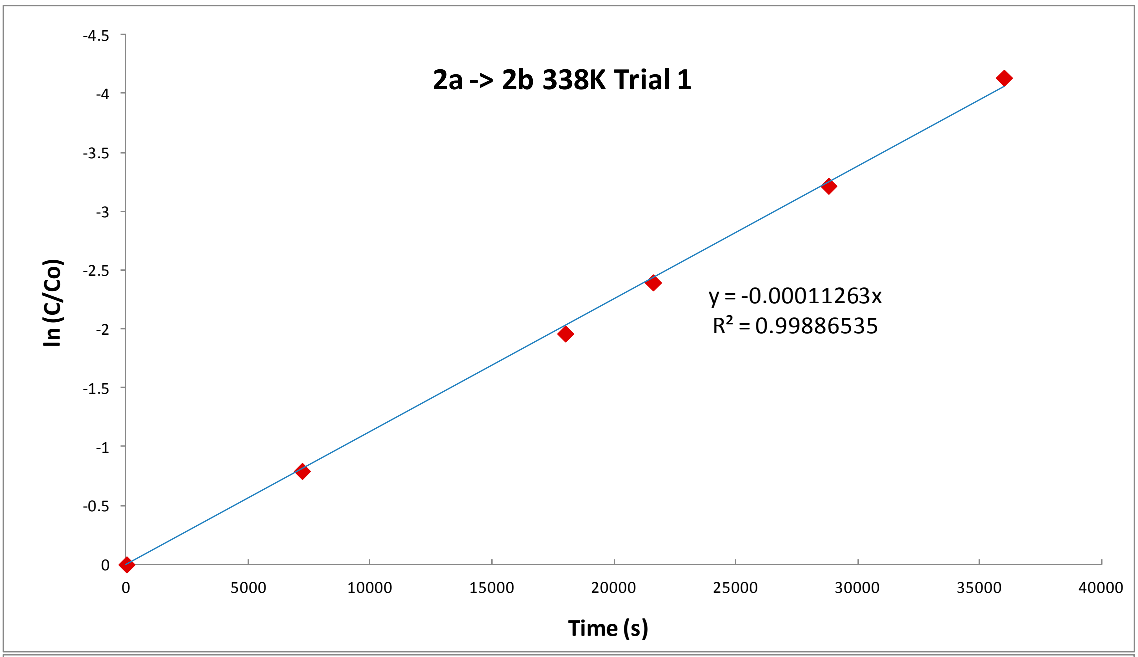
2a -> 2b 338K Trial 1
y = -0.00011263x
R² = 0.99886535
ln (C/Co)
Time (s)
-4.5
-4
-3.5
-3
-2.5
-2
-1.5
-1
-0.5
0
0
5000
10000
15000
20000
25000
30000
35000
40000

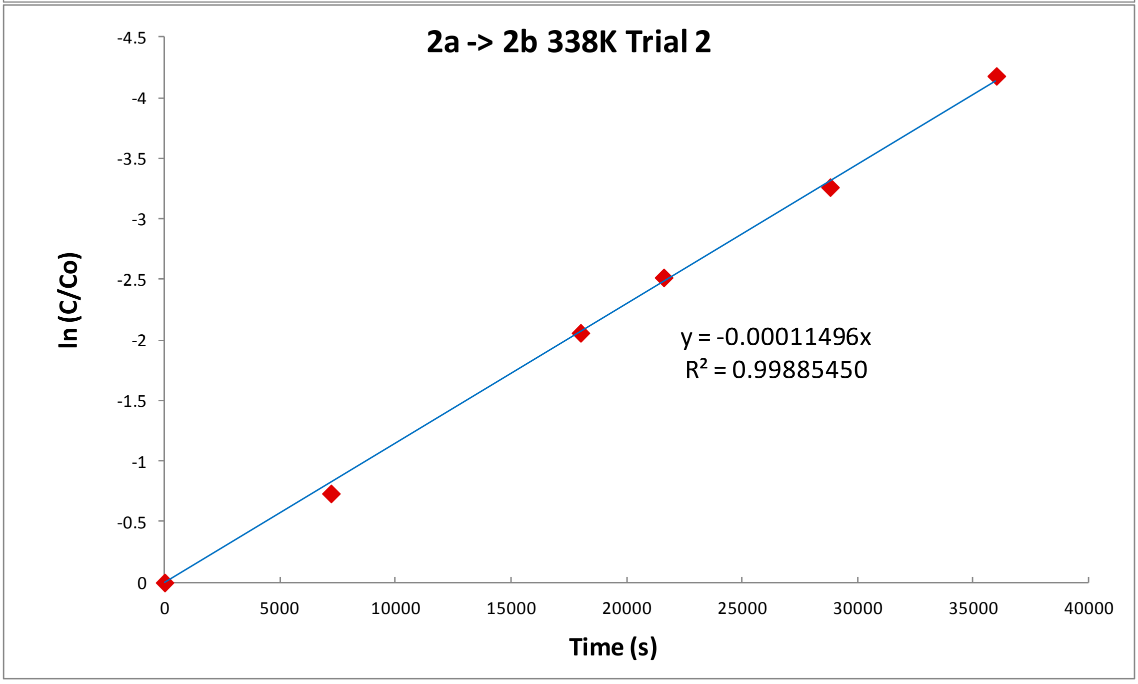
2a -> 2b 338K Trial 2
y = -0.00011496x
R² = 0.99885450
ln (C/Co)
Time (s)
-4.5
-4
-3.5
-3
-2.5
-2
-1.5
-1
-0.5
0
0
5000
10000
15000
20000
25000
30000
35000
40000

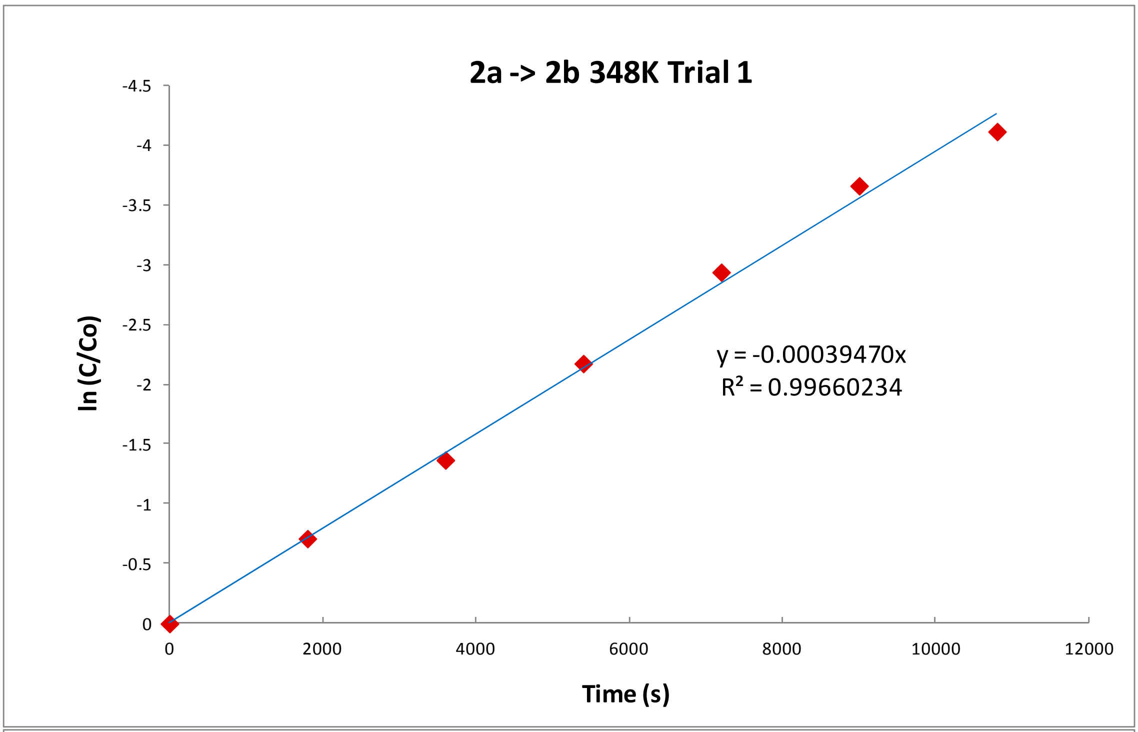
2a -> 2b 348K Trial 1
ln (C/Co)
-4.5
-4
-3.5
-3
-2.5
-2
-1.5
-1
-0.5
0
0
2000
4000
6000
8000
10000
12000
Time (s)
y = -0.00039470x
R² = 0.99660234

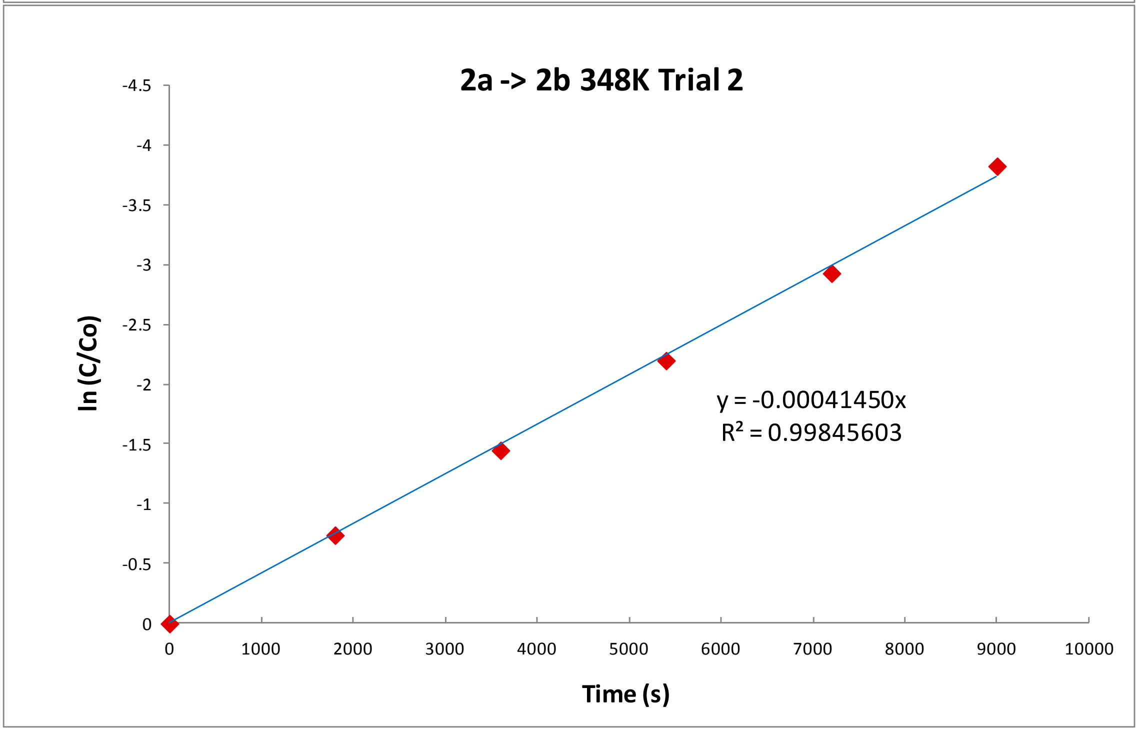
2a -> 2b 348K Trial 2
ln (C/Co)
-4.5
-4
-3.5
-3
-2.5
-2
-1.5
-1
-0.5
0
0
1000
2000
3000
4000
5000
6000
7000
8000
9000
10000
Time (s)
y = -0.00041450x
R² = 0.99845603

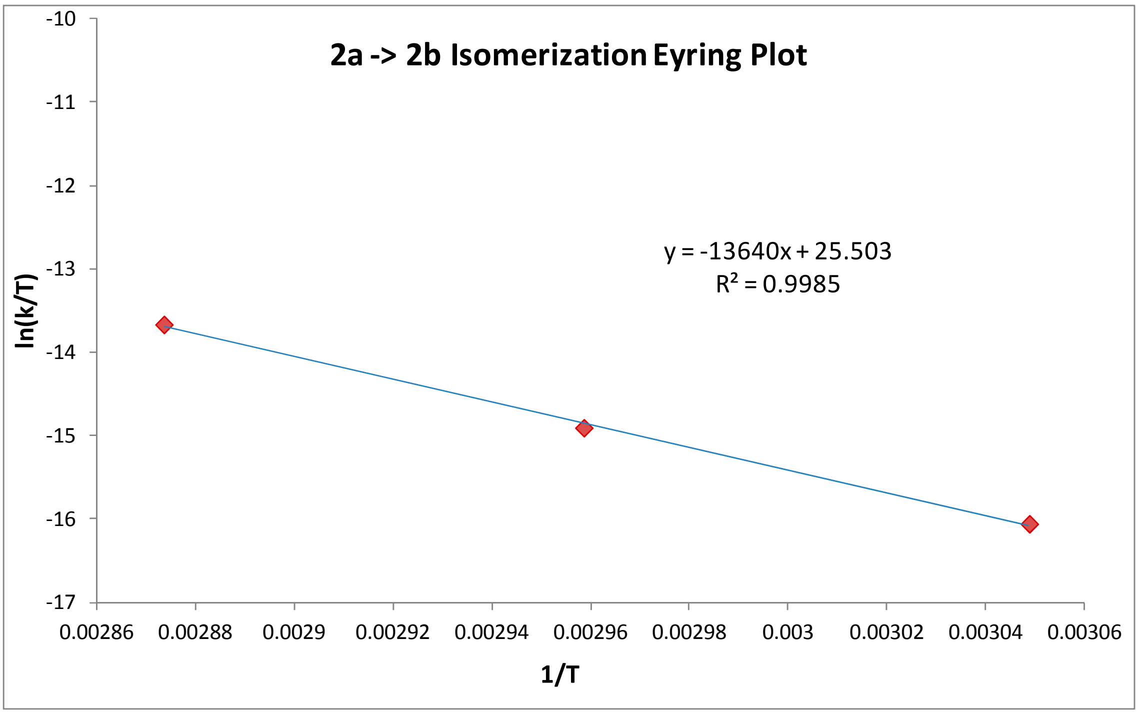

T (K)	k (s^{-1}) Trial 1	k (s^{-1}) Trial 2	k (s^{-1}) Average	ΔG‡ (kcal/mol) Average
328.0	3.288×10^{-5}	3.69×10^{-5}	3.489×10^{-5}	26.27
338.0	1.1263×10^{-4}	1.1496×10^{-4}	1.1379×10^{-4}	26.28
348.0	3.947×10^{-4}	4.145×10^{-4}	4.046×10^{-4}	26.19

Δ H‡ = 27.43 $kcalmol^{-1}$; ΔS‡ = 3.47 e.u.

Plot of rate of consumption of **4a** at 328 K, 338 K, 348 K

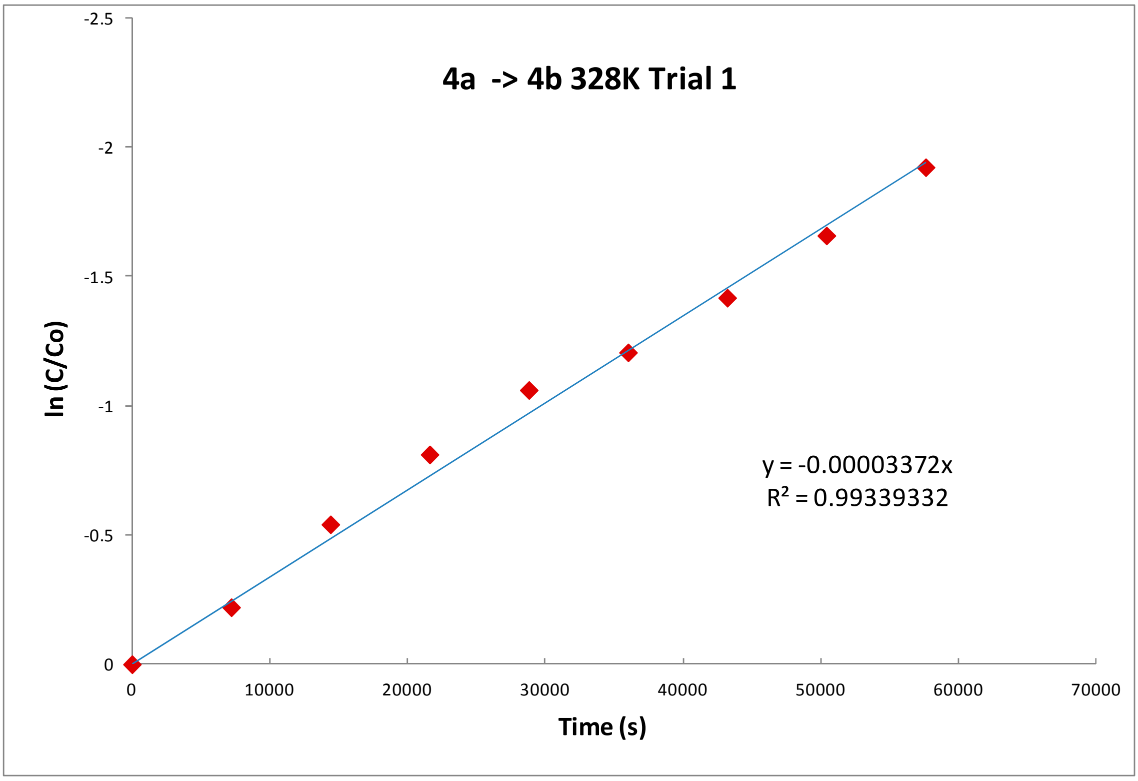

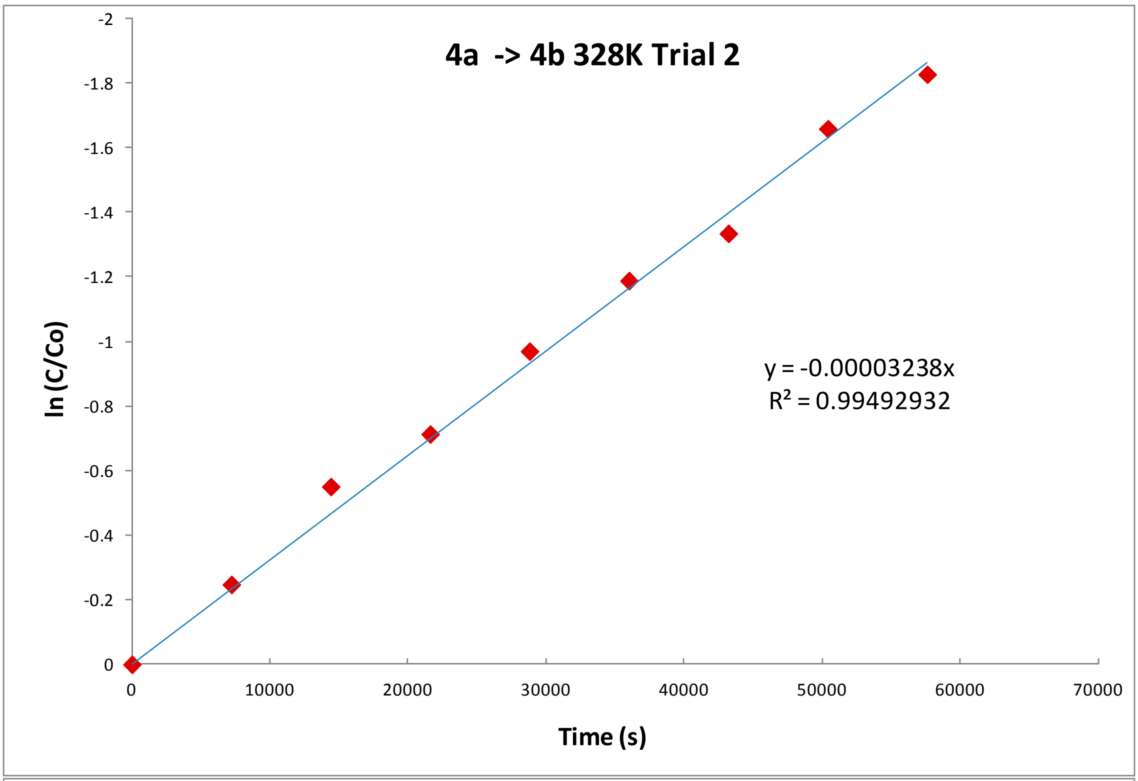
4a -> 4b 328K Trial 2
y = -0.00003238x
R² = 0.99492932
ln (C/Co)
Time (s)
-2
-1.8
-1.6
-1.4
-1.2
-1
-0.8
-0.6
-0.4
-0.2
0
0
10000
20000
30000
40000
50000
60000
70000

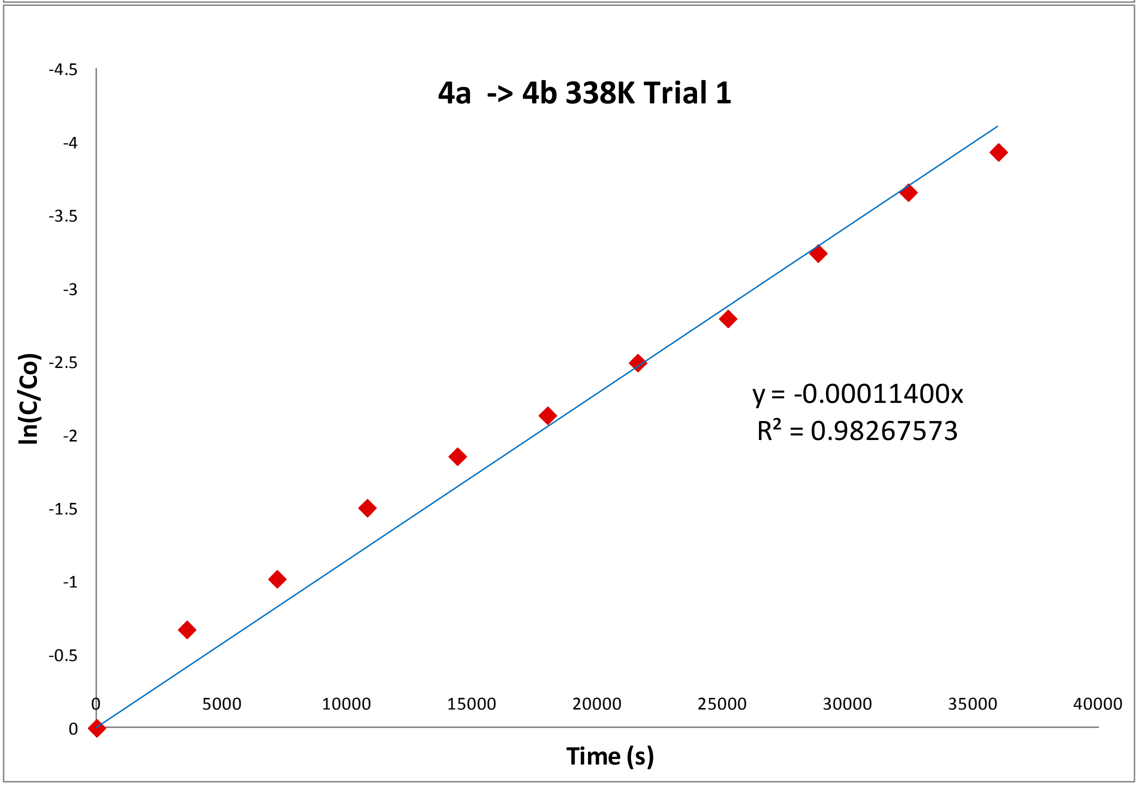
4a -> 4b 338K Trial 1
y = -0.00011400x
R² = 0.98267573
ln(C/Co)
Time (s)
-4.5
-4
-3.5
-3
-2.5
-2
-1.5
-1
-0.5
0
0
5000
10000
15000
20000
25000
30000
35000
40000

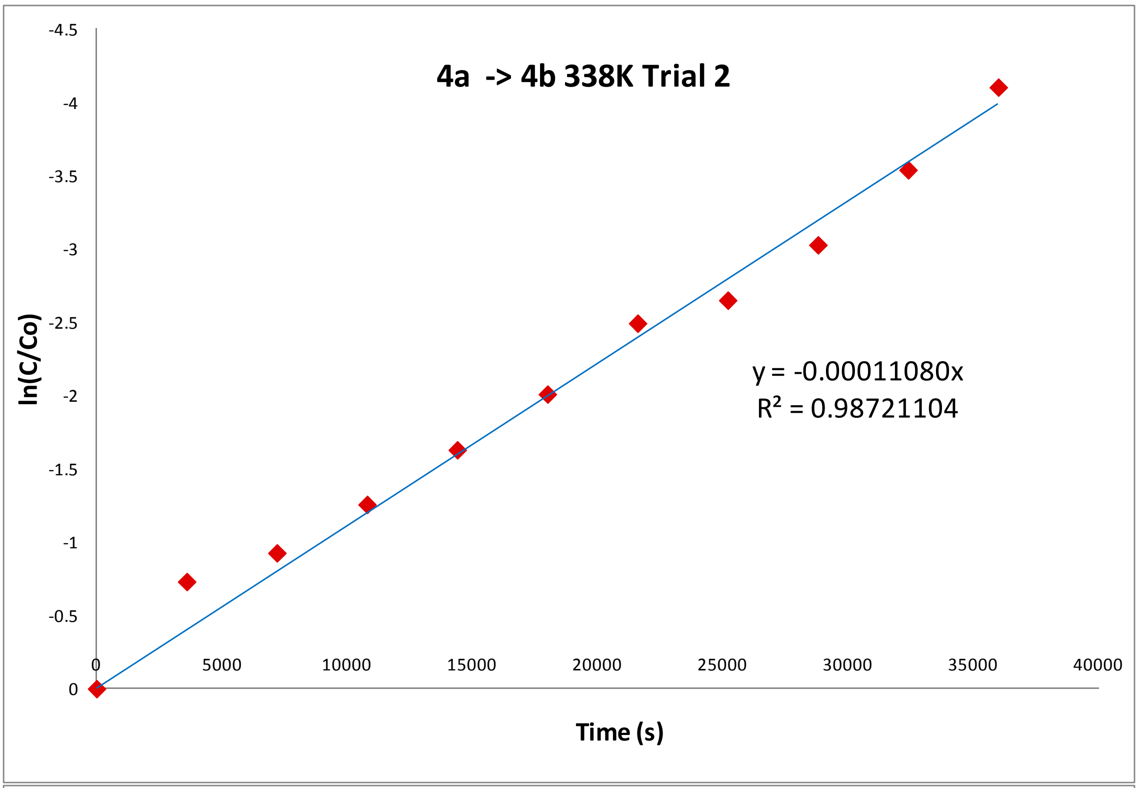
4a -> 4b 338K Trial 2
y = -0.00011080x
R² = 0.98721104
ln(C/Co)
Time (s)
-4.5
-4
-3.5
-3
-2.5
-2
-1.5
-1
-0.5
0
5000
10000
15000
20000
25000
30000
35000
40000

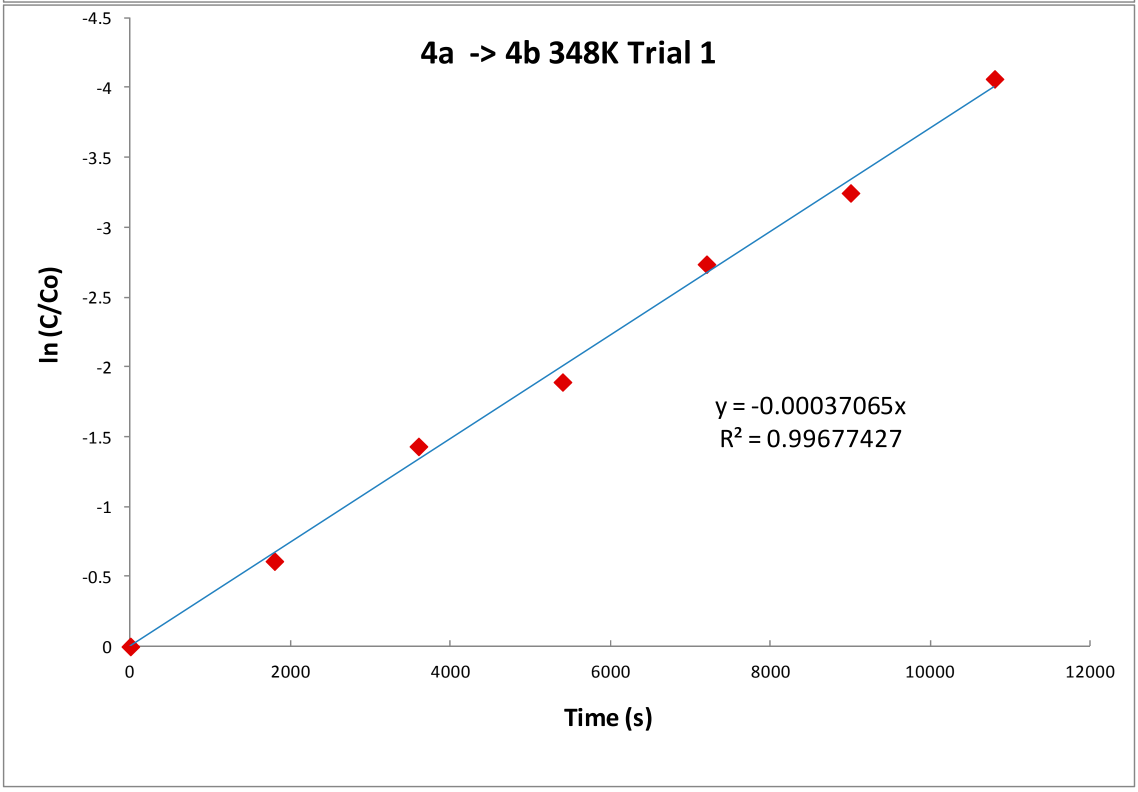
4a -> 4b 348K Trial 1
y = -0.00037065x
R² = 0.99677427
ln (C/Co)
Time (s)
-4.5
-4
-3.5
-3
-2.5
-2
-1.5
-1
-0.5
0
2000
4000
6000
8000
10000
12000

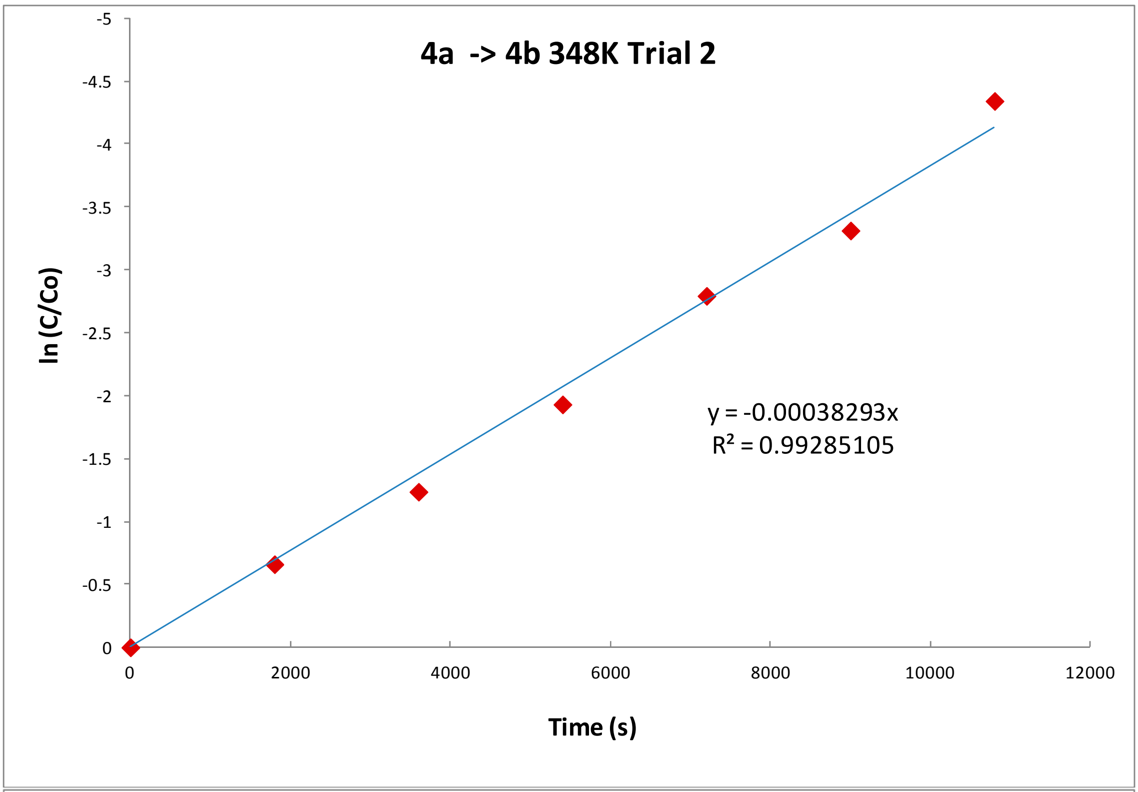

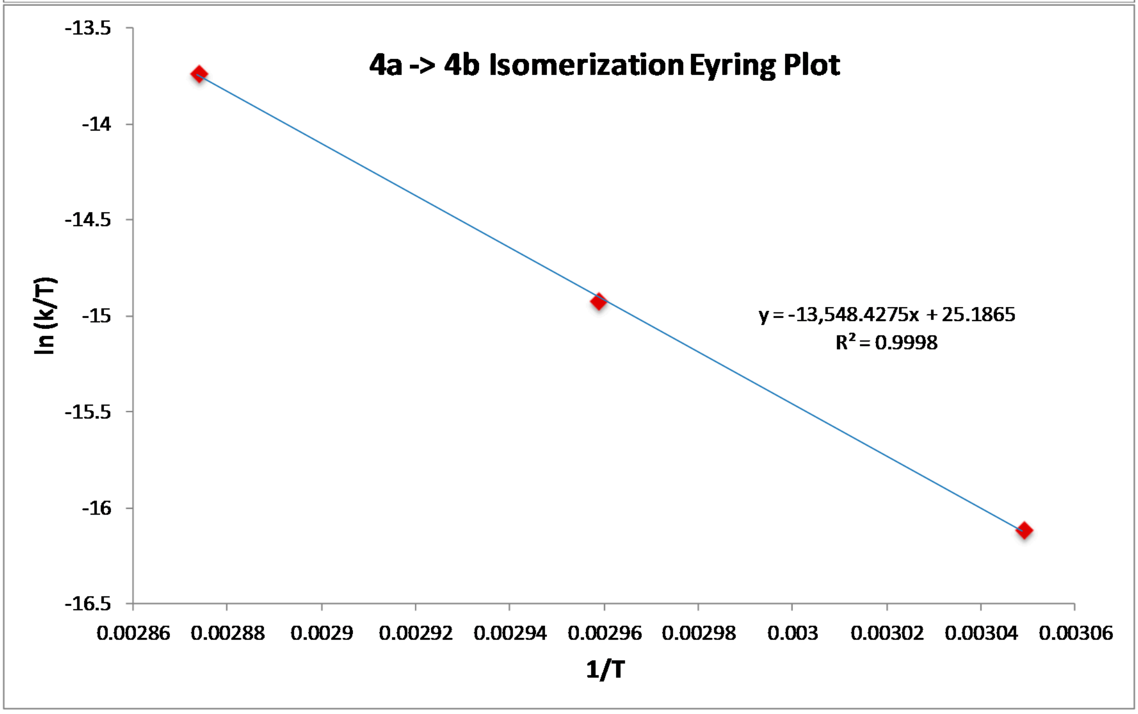

T (K)	*k* (s^{-1}) Trial 1	*k* (s^{-1}) Trial 2	*k* (s^{-1}) Average	ΔG‡ (kcal/mol) Average
328.0	3.372×10^{-5}	3.238×10^{-5}	3.305×10^{-5}	26.30
338.0	1.14×10^{-4}	1.108×10^{-4}	1.124×10^{-4}	26.30
348.0	3.7065×10^{-4}	3.8293×10^{-4}	3.7679×10^{-4}	26.24

Δ H‡ = 27.25 kcalmol^{-1}; ΔS‡ = 2.84 e.u.

Eyring Calculation:

Gradient = $-\Delta H^{‡}/R$; Intercept = $\ln(k'/h) + \Delta S^{‡}/R$

General Data Collection

Data was collected on a Bruker PLATFORM three circle diffractometer equipped with an APEX II CCD detector and operated at 1500 W (50 kV, 30 mA) to generate (graphite monochromated) Mo Kα radiation (λ = 0.71073 Å). Crystals were transferred from the vial and placed on a glass slide in Paratone® N oil. Two microscopes, a Motic SMZ-140 and an AmScope XY-PRT polarizing microscope, were used to identify a suitable specimen for X-ray diffraction from a representative sample of the material. The crystal and a small amount of the oil were collected on a MīTiGen cryoloop and transferred to the instrument where it was placed under a cold nitrogen stream (Oxford) maintained at 100 K throughout the duration of the experiment. The sample was optically centered with the aid of a video camera to insure that no translations were observed as the crystal was rotated through all positions.

A unit cell collection was then carried out. After it was determined that the unit cell was not present in the CCDC database a sphere of data was collected. Omega scans were carried out with a 20 s/frame exposure time and a rotation of 0.33° per frame. After data collection, the crystal was measured for size, morphology, and color.

Refinement Details

After data collection, the unit cell was re-determined using a subset of the full data collection. Intensity data were corrected for Lorentz, polarization, and background effects using the Bruker program APEX II. A semi-empirical correction for adsorption was applied using the program SADABS. The SHELXL-2014, series of programs was used for the solution and refinement of the crystal structure.

X-ray Characterization Data

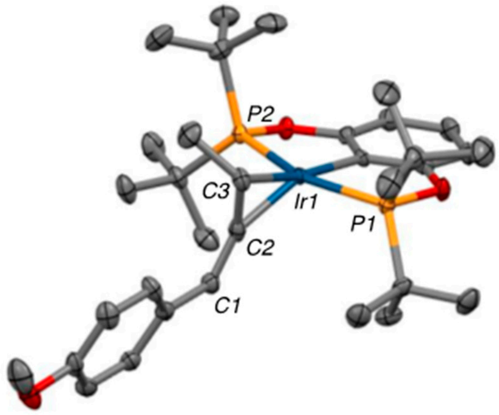

Figure S6. ORTEP diagram of **2b** with 40% probability thermal ellipsoids. Key bond lengths (Å) and bond angles (deg): C1–C2 = 1.324 (7), C2–C3 = 1.398 (7), Ir-C2 = 2.052 (5), Ir-C3 = 2.218 (4), C1–C2–C3 = 145.0 (5), P1-Ir-P2 = 157.01 (4).
